# Hspb1 protects against severe acute pancreatitis by attenuating apoptosis and ferroptosis via interacting with Anxa2 to restore the antioxidative activity of Prdx1

**DOI:** 10.7150/ijbs.84494

**Published:** 2024-02-25

**Authors:** Jun He, Xuyang Hou, Junyong Wu, Kunpeng Wang, Xiaoyan Qi, Zuxing Wei, Yin Sun, Cong Wang, Hongliang Yao, Kuijie Liu

**Affiliations:** 1 Department of General Surgery, The Second Xiangya Hospital, Central South University, Changsha, Hunan 410011, China.; 2 Department of Cardiovascular Surgery, The Second Xiangya Hospital, Central South University, Changsha, Hunan 410011, China.; 3 Department of Pharmacy, The Second Xiangya Hospital, Central South University, Changsha, Hunan 410011, China.; 4 Department of General Surgery, Taizhou Central Hospital (Taizhou University Hospital), Taizhou University, Taizhou, Zhejiang, China.; 5 Institute of Pharmaceutical Pharmacology, University of South China, Hengyang, Hunan, China.

**Keywords:** Hspb1, Severe acute pancreatitis, Apoptosis, Ferroptosis, Anxa2, Prdx1

## Abstract

Acute pancreatitis (AP) is a common abdominal disease that typically resolves on its own, but the mortality rate dramatically increases when it progresses to severe acute pancreatitis (SAP). In this study, we investigated the molecular mechanism underlying the development of SAP from AP. We utilized two SAP models induced by pancreatic duct ligation and caerulein administration. Transcriptomic and proteomic analyses were subsequently performed to determine the mRNA and protein expression profiles of pancreatic samples from SAP and AP model and normal mice.

To explore the role of Hspb1 in SAP, we used Hspb1 knockout (KO) mice, a genetically engineered chronic pancreatitis strain (T7D23A), Anxa2 KO mice, and acinar cell-specific Prdx1 knockout mice. Additionally, various in vivo and in vitro assays were performed to elucidate the molecular events and direct targets of Hspb1 in acinar cells. We found that Hspb1 expression was upregulated in AP samples but significantly reduced in acinar cells from SAP samples. KO or inhibition of Hspb1 worsened AP, while AAV8-Hspb1 administration mitigated the severity of SAP and reduced remote organ damage in mice. Furthermore, AAV8-Hspb1 treatment prevented the development of chronic pancreatitis. We found that KO or inhibition of Hspb1 promoted acinar cell death through apoptosis and ferroptosis but not necroptosis or autophagy by increasing reactive oxygen species (ROS) and lipid ROS levels. Mechanistically, Hspb1 directly interacted with Anxa2 to decrease its aggregation and phosphorylation, interact with the crucial antioxidant enzyme Prdx1, and maintain its antioxidative activity by decreasing Thr-90 phosphorylation. Notably, the overexpression of Hspb1 did not have a protective effect on acinar-specific Prdx1 knockout mice.

In summary, our findings shed light on the role of Hspb1 in acinar cells. We showed that targeting Hspb1/Anxa2/Prdx1 could serve as a potential therapeutic strategy for SAP.

## Introduction

Acute pancreatitis (AP) is a common acute abdominalgia that typically resolves on its own; however, the complication and mortality rates substantially increase if the disease progresses to severe acute pancreatitis (SAP)^1^, ^2^. Although most AP patients experience limited symptoms that resolve spontaneously within days, patients with SAP have a mortality rate of up to 20%^3^. The severity of AP is largely determined by the degree of pancreatic damage and the immune response. The etiological factors, such as alcohol abuse or pancreatic duct blockage, initially activate pancreatic digestive enzymes within acinar cells and trigger acute inflammation mainly mediated by neutrophils and macrophages^4^-^6^. During the progression of AP into SAP, acinar cells swell and undergo apoptosis, leading to excessive programmed cell death^7^, ^8^. Exploring the molecular mechanism underlying this process will be beneficial for identifying novel therapeutic options for the treatment of SAP.

Fast-developing multimodal omics technologies have allowed the comprehensive analysis of molecular variations among different conditions. Recently, the integration of transcriptomic and proteomic data has been used to determine the molecular signatures involved in many diseases, including cancer, inflammatory diseases and diabetes^9^-^12^. However, multimodal omics studies of AP are lacking, and we explored the essential molecules involved in the process of AP progression into SAP through transcriptomics and proteomics analysis. Hspb1 (also known as heat shock protein 27) is a member of the small heat shock protein family and functions as a chaperone by interacting with protein clients to maintain protein solubility^13^, ^14^. Hspb1 is broadly expressed in various tissues in humans and mice and is extensively implicated in many processes, such as antioxidation, proteasomal degradation of misfolded proteins, and neuroprotection^15^-^17^. Previous studies demonstrated that overexpression of Hspb1 in the pancreas could ameliorate acinar cell death in a caerulein-induced AP model^18^-^20^. However, evidence verifying the role of Hspb1 in SAP and its exact mechanism is lacking. Hspb1 was shown to inhibit intracellular reactive oxygen species (ROS) production, promote the release of cytochrome c and activate the caspase cascade in the pancreas^21^, ^22^. Reports have also suggested that Hspb1 has an antagonistic effect on ferroptosis by reducing the iron-mediated production of lipid ROS in cancer cells^23^, ^24^. Apoptosis and ferroptosis are both involved in the development of SAP^25^, ^26^. Thus, we attempted to determine in detail the role of Hspb1 in SAP.

In the present study, we first compared the RNA and protein expression of genes among normal pancreatic, AP and SAP samples by transcriptomics and proteomics and found that Hspb1 was highly upregulated in AP but attenuated in SAP. Regardless of whether pharmacological inhibition or knockout of Hspb1 exacerbated AP, pancreatic duct administration of AAV8 (adeno-associated virus 8)-Hspb1 significantly alleviated pancreatic damage, neutrophil and macrophage infiltration, and remote damage to the lung and intestine in the two SAP models. We modified a genetically engineered chronic pancreatitis (CP) model by inserting the p.D23A mutation into exon 2 of the trypsinogen 7 precursor and demonstrated that overexpression of Hspb1 blocked the development of CP. Mechanistically, inhibition of Hspb1 in acinar cells promoted apoptosis and ferroptosis but did not affect autophagy or necroptosis. Hspb1 acted as a chaperone to facilitate the interaction between Anxa2 and Prdx1 to inhibit the tyrosine phosphorylation of Prdx1 and maintain its antioxidative activity in acinar cells challenged with SAP. Finally, we used acinar-specific Prdx1 knockout mice and conclusively demonstrated that Prdx1 was a critical downstream target of Hspb1 in acinar cells during SAP.

## Materials and methods

### Reagents

J2 (GC38503), DHE (GC30025), and C11 BODIPY 581/591 (GC40165) were purchased from Glpbio, America. Erastin (S7242), caerulein (C9026) and N-acetylcysteine (S1623) were purchased from Selleck Chemicals, Japan. Soybean trypsin inhibitor (T6522), taurocholate acid (T4009), and L-arginine (A5131) were purchased from Sigma‒Aldrich, America. J2 (25 mg/kg) was administered via intraperitoneal injection for three consecutive days before establishing the AP model.

### SAP mouse models

All animal protocols were reviewed and approved by the Institutional Animal Care and Use Committee (IACUC) of Central South University, and we rigorously complied with all the relevant ethical requirements. Wild-type mice (C57BL/6J background) were purchased from Hunan SJA Laboratory Animal Co., Ltd. For the caerulein-induced SAP model, 6- to 8-week-old male mice were fasted for 12 hours and received 12 hourly intraperitoneal injections of caerulein (50 μg/kg diluted in sterile saline). The mice were sacrificed 24 hours after the first injection, after which the blood, pancreas, lung and intestine were collected for further analysis. The protocol for establishing the pancreatic duct ligation-induced SAP model was described in our previous report^27^. Six- to eight-week-old male mice were fasted for 12 hours, after which ketamine and xylazine were used as anesthetic agents. After the duodenum was exposed, a blunted end cannula was passed through the papillary side, 50 µl of methyl blue was injected into the duct as an indicator, and the main duct was carefully ligated without influencing the bile duct. The next day, three doses of caerulein (50 µg/kg body weight) were injected at one-hour intervals. Blood, pancreas, lung and intestine were collected from the SAP model mice after 2 days.

The Tryp7^mut^ strain was generated on the C57BL/6J background and created by CRISPR/Cas-mediated genome engineering at Cyagen, China. The p.D23A mutation (GAC to GCG) was introduced into exon 2 of the mouse 2210010C04Rik gene (trypsinogen 7 precursor) by homology-directed repair. The targeting vector was coinjected into fertilized eggs with the Cas9 protein and gRNA, after which the pups were genotyped via PCR and sequencing analysis. After the mice were crossed with E2a-Cre mice (purchased from Cyagen, Inc.), Typ7^mut^; E2a-Cre (named the T7D23A strain) mice were obtained. Genomic DNA was extracted from the tails of 2-week-old mice, and genotypic identification of Typ7^mut^ was carried out with the following primers: wild-type F, TGAGTCTTGCTAAATCGTCCAAGC; wild-type R, ATCTTGGCAGGCGATAAGTTGTTG; mutated type F, TGAGTCTTGCTAAATCGTCCAAGC; mutated type R, ACTTGTGTAGCGCCAAGTGC.

Prdx1^fl/fl^ (purchased from Cyagen, Inc.) were bred with Ptf1α-Cre mice (purchased from the Shanghai Model Organisms Center, Inc.) to generate acinar cell-specific Prdx1 knockout (Prdx1-cKO) mice. The flox and knockout mice were littermates and cage mates, respectively. The Hspb1 KO strain was generated on the C57BL/6J background and created by CRISPR/Cas-mediated genome engineering at Cyagen, China. The Hspb1 gene is located on chromosome 5, and exons 1-3 are the targeted deletion regions. The Cas9 protein and gRNA of Hspb1 were coinjected into fertilized eggs, and the offspring were identified via PCR and sequencing analysis. For regular genotyping, DNA was extracted from the tails of 2-week-old mice. The genotypic identification of Prdx1 floxed mice was carried out with the following primers: forward, CCAGCTTCATGAGTAGGTAGGATT; reverse, GTGTGCTCACCAGTGCATATTAGA.

### Clinical specimens

Patient samples were collected from the Second Xiangya Hospital, Central South University, Changsha, China. The use of clinical samples was in accordance with the principles of the ethical guidelines of the 1975 Declaration of Helsinki and was approved by the institutional review board of the institute.

### Histological analysis

The pancreas, lung and intestine were fixed with 4% paraformaldehyde and embedded in paraffin, and 4 µm sections were cut with a microtome. H&E staining of these tissues was performed after deparaffinization and dehydration of the slides. Images were acquired with a Zeiss microscope.

### Analysis of amylase and lipase activity

Plasma was obtained from blood with 20 μl of 0.5 M EDTA by centrifugation at 3000 rpm/min for 10 min. The trypsin and lipase activities in plasma were measured by a clinical analysis system. Then, 100 μl of plasma was sent to another lab for analysis. To determine amylase activity, a kit that utilizes the substrate EPS was employed, and a kit that uses the substrate methyl triazine was employed to determine lipase activity. The signals were determined with an AU1000 system (Beckman, America).

### Isolation of primary acinar cells

The protocol for isolating acinar cells from mice was based on a mechanical and enzymatic dissociation technique, as described in our previous report^27^. In brief, 6- to 8-week-old mice were sacrificed by CO_2_ asphyxiation, and the pancreas was cut and immersed in Waymouth's medium. The pancreas was cut into approximately 1 cm pieces and incubated in HBSS 1x buffer with 200 U/ml collagenase IA, 0.25 mg/ml trypsin inhibitor and 0.2% FBS on a shaker (80 rpm/min) at 37 °C for 20 min. Then, the pancreas was triturated slightly by passing through a 1 ml pipette tip 10 times and filtering through a 100 µm filter. After two washes with PBS, the acinar cells were resuspended in complete medium (Waymouth's medium containing 2.5% FBS, 1% penicillin/streptomycin, 0.25 mg/ml trypsin inhibitor, and 25 ng/ml recombinant human epidermal growth factor) and cultured at 37 °C in a humidified incubator containing 5% CO_2_.

### Cell culture

The 266-6 cell line was purchased from Bhcell Biotec and cultured in Dulbecco's modified Eagle medium (DMEM; Gibco, America) supplemented with 10% FBS at 37 °C, 95% humidity, and 5% CO_2_. To mimic AP in vitro, we added 5 mM taurocholate acid and incubated the mixture for 24 h. The AR42J cell line was obtained from Sunncell Biotec and cultured in DMEM supplemented with 20% FBS at 37 °C, 95% humidity, and 5% CO_2_. Cell line identification was performed by the corresponding providers, and mycoplasma testing was negative for contamination.

### Plasmids and siRNA transfection

The plasmids used were generated by Sangon Biotech (Shanghai) and transfected with Lipofectamine^TM^ 2000 reagent. siRNAs were designed with the siCatchTM siRNA design system and synthesized by RiboBio Biotech (Guangzhou, China). Transfection (100 pmol/ml) was performed using Lipofectamine^TM^ RNAiMAX reagent.

### Immunofluorescence (IF) and immunohistochemistry (IHC)

Freshly prepared or paraffin-embedded samples were cut into 4 μm-thick sections. Most procedures used were the same for both types of tissues; thus, we only described the protocols used for the paraffin-embedded tissue sections. For IF staining, the sections were heated to 60 °C for 1 h, deparaffinized by rinsing in xylene and dehydrated in various concentrations of ethanol. After antigen retrieval by sodium citrate buffer (10 mM sodium citrate and 0.05% Tween 20 at pH 6.0) at 96 °C for 20 min, the following primary antibodies were added to the slides: anti-amylase (Santa Cruz Biotechnology, 1:50), anti-insulin (Santa Cruz Biotechnology, 1:100), anti-Hspb1 (Cell Signaling Technology, 1:100), anti-GPX4 (Santa Cruz Biotechnology, 1:50), anti-SLC3A2 (Santa Cruz Biotechnology, 1:50), anti-SLC7A11 (Santa Cruz Biotechnology, 1:50), anti-cleaved caspase 3 (Cell Signaling Technology, 1:100), anti-Anxa2/p-Anxa2 (Santa Cruz Biotechnology, 1:50), anti-CD68 (Cell Signaling Technology, 1:100) and anti-Prdx1/p-Prdx1 (Cell Signaling Technology, 1:100). The samples were washed twice with PBS to remove unbound antibodies and incubated with Alexa Fluor 488- or 594-conjugated secondary antibodies for 45 min at room temperature. Then, the sections were washed with PBS and mounted with DAPI containing antifade reagent (Invitrogen, USA). Images were captured using a Zeiss microscope.

For IHC, the sections were deparaffinized by heating to 60 °C for 1 hour and dehydrated by rinsing in xylene and various concentrations of ethanol. IHC was conducted using an IHC kit (ZSGB-BIO, China) following the manufacturer's instructions. After antigen retrieval with sodium citrate buffer, primary antibodies were added, and the cells were cultured overnight. The sections were rinsed in PBS for 5 min, and 3% H_2_O_2_ was added to reduce endogenous peroxidase activity for 20 min. Then, the sections were incubated with response enhancer (provided by the kit) for 20 min and washed twice with PBS. The sections were incubated with secondary antibody at 37 °C for 20 min. The sections were rinsed and incubated with diaminobenzidine working solution (20x storage solution) for 3-6 min. The sections were washed with ddH_2_O for 10 min, and the nuclei were stained with hematoxylin for 5 min. After dehydration, the sections were mounted with neutral resins, and images were captured using a Zeiss microscope.

### Delivery of adeno-associated virus 8 (AAV8) to the pancreas by intraductal administration

To express specific genes in the pancreas, we used AAV8 to deliver targeting sequences via pancreatic duct infusion. The protocol was described in our previous work^27^. Briefly, the mice were anesthetized, the abdomen was opened at the midline, and the pancreatic duct was exposed. Then, 150 μl of PBS with 3x 10^12^ vector genomes of AAV8 was prepared for each mouse and administered via an infusion syringe pump. A 31 G needle was used to penetrate the duodenum opposite the sphincter of Oddi, and the ampulla was clamped around the catheter and the common bile duct. Then, the pump was turned on, and the pancreas was infused at a flow rate of 6 μl/min for a total of 150 μl. After completing the infusion, the serrefines were removed, and the catheter was gently withdrawn. The hole in the duodenum was closed, the intestines were returned to the normal position in the abdomen, and the incision was sutured with 6-0 sutures. Two weeks after infusion, the mice exhibited demonstrable results, as presented in this work.

### Coimmunoprecipitation (co-IP)

For the co-IP assay, the Pierce^TM^ Co-IP Kit (Thermo Fisher, America) was used following the manufacturer's instructions. Primary acinar cells were treated as indicated, and HEK293T cells were cotransfected with the indicated plasmids and then lysed with cold IP lysis buffer supplemented with 1x protease and phosphatase inhibitor cocktail (Thermo Fisher, America) for 5 min with periodic mixing. The tube was centrifuged at 13000 × g for 10 min to remove the cell debris. A 500 µl IP sample was mixed with 2 μg of the corresponding antibody and incubated at 4 °C overnight. Then, 25 μl of Pierce Protein A/G agarose beads was added, mixed well, and incubated for 1 hour at room temperature. Then, the beads were washed with cold lysis buffer 4 times and centrifuged to remove the unbound proteins. Then, 50 μl of sample-buffer elution buffer was added, the mixture was boiled at 100 °C for 5 min and centrifuged to collect the eluate. The samples were subjected to mass spectrometry analysis (Novogene, China) or SDS‒PAGE.

### Transferase-mediated dUTP nick-end-labeling (TUNEL) assay

TUNEL assays were conducted with a TUNEL kit (Beyotime, China) following the manufacturer's instructions. Briefly, tissue sections were heated to 60 °C for 1 hour, deparaffinized with xylene and dehydrated with ethanol. The proteins in the sections were removed by incubation with protease K for 30 min at 37 °C. After two washes with PBS, TUNEL reaction solution was added, and the samples were incubated for 1 hour at 37 °C. Then, the sections were washed twice and mounted with DAPI containing Antifade reagent (Invitrogen, USA). Images were captured using a Zeiss microscope.

### Flow cytometry analysis

Then, 500 µl of blood was collected, 5 ml of ACK lysis buffer (Gibco, America) was added, and the mixture was incubated at room temperature for 10 min. After centrifugation at 1000x g for 5 min, the cells were collected and resuspended in PBS. The number of cells was counted, and 1x 10^6^ cells were transferred to a new EP tube. An antibody cocktail (viability staining, CD11b, Ly6G and CD45) was added, and the samples were incubated for 45 min at room temperature. All of the antibodies used were purchased from BD Pharmingen. The cells were centrifuged at 1000x for 5 min and then fixed with 4% paraformaldehyde. The samples were analyzed by a flow cytometer (BD Biosciences). The data analysis was carried out using FlowJo (version 11).

### Lipid peroxidation malondialdehyde (MDA) assay

For the MDA assay, a kit was purchased from Beyotime (China) and used according to the manufacturer's instructions. The tissues were lysed and centrifuged at 10000x g for 10 min. The protein concentration was measured by a BCA assay. TBA working solution (TBA, dilution, and antioxidant) was prepared, mixed with the supernatant of the tissues and boiled for 15 min. After the samples were cooled to room temperature, the samples were centrifuged at 1000x g for 10 min, after which the absorbance of the supernatant was measured at a wavelength of 532 nm.

### Cytokine measurement in bronchoalveolar lavage fluid (BALF)

To collect BALF samples, 0.8 ml of saline solution was injected into the lung. After centrifugation at 3000x g for 10 min, the supernatant was collected. The levels of TNFα, IL1β, and IL6 were measured by corresponding ELISA kits purchased from ABclonal (China). The procedures were performed according to the manufacturer's instructions.

### *In vivo* permeability using FITC-dextran

Then, 100 mg/kg 4 kDa FITC-dextran (Sigma‒Aldrich, America) was prepared and gavaged into mice as indicated. After 1.5 hours, the mice were sacrificed, and whole blood was collected via centrifugation at 3000 rpm for 10 min to obtain plasma. Next, 100 µl of plasma from each mouse was added to a 96-well plate, and the fluorescence signal (520 nm wavelength) was measured with a microplate spectrofluorometer (Thermo Fisher, USA). The in vivo permeability is shown as the relative percentage of the FITC signal after subtracting the plasma signal from mice that were not gavaged with FITC-dextran.

### RNA extraction and quantitative real-time PCR (qRT‒PCR)

The RNA was extracted from tissues and cells as described in our previous report. The RNA purity and concentration were analyzed using a Nanodrop 1000 spectrophotometer (Thermo Fisher, America). cDNA was synthesized using a high-capacity cDNA reverse transcription kit (Life Tec, America). A 2X Universal SYBR Green Fast qRT‒PCR Mix (ABclonal, China) was used for qRT‒PCR.

The primers used were as follows: Hspb1, 5'-CTCACAGTGAAGACCAAGGAAG-3' (forward) and 5'-GAGAGATGTAGCCATGTTCGTC-3' (reverse); β-actin, 5'-GAGGTATCCTGACCCTGAAGTA-3' (forward) and 5'-CACACGCAGCTCATTGTAGA-3' (reverse); 16S rRNA, 5'-TCGTCGGCAGCGTCAGATGTGTATAAGAGACAGCCTACGGGNGGCWGCAG-3' (forward) and 5'-GTCTCGTGGGCTCGGAGATGTGTATAAGAGACAGGACTACHVGGGTATCTAA-TCC-3' (reverse).

### Protein extraction and western blot analysis

Samples from tissues or cells were lysed in RIPA buffer supplemented with 1x protease and phosphatase inhibitor cocktail (Thermo Fisher, America) and incubated on ice for 30 min. After centrifugation at 14000 × g for 10 min at 4 °C, the supernatant was collected to determine the protein concentration by BCA assay. The samples were prepared for electrophoresis, followed by transfer onto PVDF membranes. The primary antibodies used in this work were as follows: anti-ZO-1 (Santa Cruz Biotechnology), anti-p-MLKL (Cell Signaling Technology), anti-p-RIP1 (Cell Signaling Technology), anti-cleaved/full-length caspase 9 (Cell Signaling Technology), anti-cleaved/full-length caspase 3 (Cell Signaling Technology), anti-GPX4 (Santa Cruz Biotechnology), anti-SLC3A2 (Santa Cruz Biotechnology), anti-SLC7A11 (Santa Cruz Biotechnology), anti-ANXA2 (ABclonal), anti-His-tag (ABclonal), anti-HA-tag (ABclonal), anti-HSPB1 (Cell Signaling Technology), and anti-PRDX1 (Cell Signaling Technology). Secondary antibodies were purchased from ABclonal. The membranes were visualized via the chemiluminescence method.

### Dihydroethidium (DHE) and C11 BODIPY 581/591 fluorescent probes

DHE (Glpbio, America) was used to evaluate intracellular ROS in vitro and in vivo. For staining, 5 μM DHE was added to the tissues for 10 min. After the samples were washed with PBS 2 times, the tissue sections were mounted with DAPI containing antifade reagent. For in vivo application, 200 μl of DHE at a concentration of 50 μM was infused into the pancreas through the pancreatic duct, and the pancreas was collected after 30 min. The fresh pancreas was cut into 4 μm-thick sections using a freezing microtome (Leica, Germany). Then, 2.5 μM C11 BODIPY 581/591 was added to the sections for staining for 30 min. Images were captured using a Zeiss microscope.

### Scanning electron microscopy analysis

Pancreatic tissues from the mice were cut into 1 mm^3^ pieces, fixed in 2.5% glutaraldehyde at room temperature for 1 hour and further fixed at 4 °C overnight. Then, the samples were subjected to electron microscopy at Xiangya Hospital, and the following analysis was performed according to the standard procedures of the institute.

### RNA sequencing

Total RNA was extracted from the pancreas as indicated. The amounts and integrity of the RNA were evaluated by an RNA Nano 6000 Assay Kit. mRNA purification was performed by poly-T olig-attached magnetic beads, and first-strand cDNA and second-strand cDNA were synthesized by M-MuLV Reverse Transcriptase and DNA Polymerase I, respectively. Libraries were sequenced by the Illumina NovaSeq 6000 system. After deleting the reads with impurities, the clean data were mapped to the reference genome. FeatureCounts was used to count the reads mapped to various genes, and FPKM (fragments per kilobase of transcript sequence per million) was used to estimate the gene expression level. DESeq2 was used to evaluate the differentially expressed genes. Adjusted p values less than 0.05 were considered to indicate statistical significance.

### Proteomic analysis

Pancreatic tissues were collected, and protein was extracted in lysis buffer with 1x protease and phosphatase inhibitor cocktail (Thermo Fisher, America). After centrifugation, the supernatant was digested with trypsin, and the resulting peptides were labeled with TMT. The samples were subsequently fractionated using a C18 column on a Rigol L3000 HPLC system. Then, the samples were subjected to LC‒MS/MS analysis. The data analysis was performed at Novogene, China. Proteins with a p value <0.05 were identified as differentially expressed proteins.

### Generating Hspb1 KO cell

To generate Hspb1 KO cells based on 266-6 cells using CRISPR-Cas9 technology, the following methods were employed. Three guide RNAs (gRNAs) were designed to target specific sequences within the Hspb1. The gRNAs were then cloned into a CRISPR plasmid along with the Cas9 nuclease gene. The constructed CRISPR plasmid was transfected into the 266-6 cells using lentivirus vectors. Following transfection, cells were subjected to 5 ug/ml puromycin for 72 hours. After selection, individual clones were isolated and expanded. To confirm the generation of Hspb1 KO cells, Western blotting was performed, and the most efficient KO cell line was selected for subsequent experiments.

### Statistical analysis

All of the data are expressed as the mean ± standard error and were analyzed using GraphPad Prism 8. The significance of differences between 2 groups was analyzed by unpaired Student's t test (using a parametric test when the data had a normal distribution and homoscedasticity otherwise using a nonparametric test), and one-way analysis of variance was used to determine the differences among multiple groups. A p value of < 0.05 was considered to indicate statistical significance.

## Results

### Transcriptomic and proteomic analyses revealed that Hspb1 was upregulated in AP but attenuated in SAP

**Figure 1:**
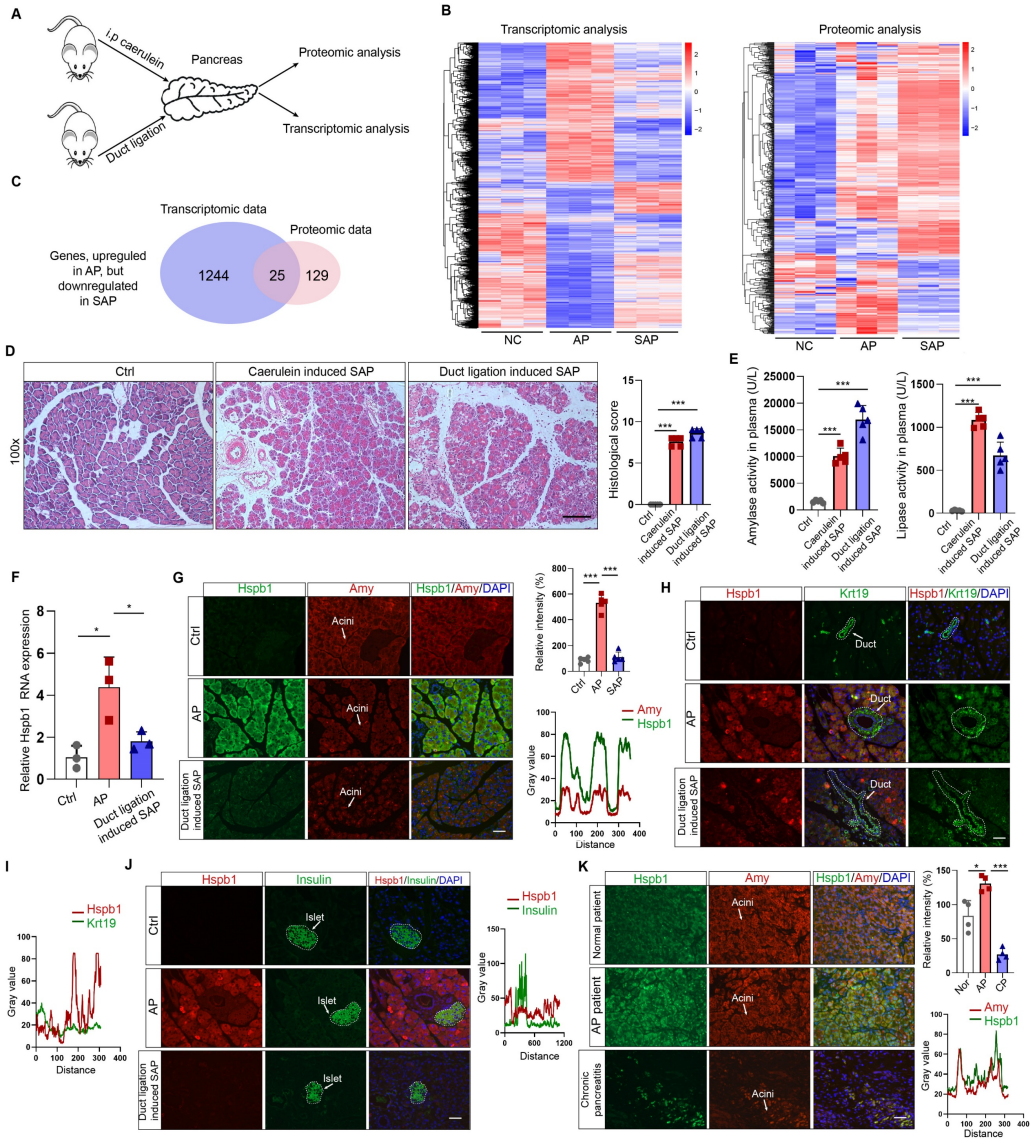
** Transcriptomic and proteomic analyses revealed that Hspb1 was upregulated in AP but attenuated in SAP.** (A) Schematic of the transcriptomic and proteomic procedures. The pancreases of the normal, AP (intraperitoneal injection of caerulein every 8 hours), and SAP (pancreatic duct ligation) groups were collected. (B) Heatmaps showing the differentially expressed genes among the normal, AP and SAP samples according to transcriptomics and proteomics. Three biological replications were performed for each group. Clustering analyses were performed by hierarchical clustering of genes with similar expression patterns. (C) The genes identified by both transcriptomics and proteomics were significantly upregulated in AP but attenuated in SAP. (D-E) H&E staining of the pancreas and amylase and lipase activity in the plasma of the caerulein-induced and duct ligation-induced SAP models (n=5). The histological scoring of the pancreas was performed by a pathologist using the standards described in the methodology section. Scale bar, 100 μm. (F) Relative expression of Hspb1 RNA in normal, AP and duct ligation-induced SAP pancreatic tissues. (G) Representative images of Hspb1 (green) in acinar cells (marked by amylase, red) in the normal, AP and duct ligation-induced SAP models (n=5). The fluorescence signal of Hspb1 was quantified using ImageJ software (upper), while the colocalization of Hspb1 and amylase in the AP group was also analyzed using ImageJ (lower). Scale bar, 50 μm. (H-I) Representative images of Hspb1 (red) in duct cells (marked by Krt19, green) from the normal, AP and duct ligation-induced SAP models (n=5). The fluorescence signal of Hspb1 was quantified using ImageJ software (upper), while the colocalization of Hspb1 and Krt19 in the AP group was also analyzed using ImageJ (lower). Scale bar, 50 μm. (J) Representative images of Hspb1 (red) in islet cells (marked by insulin, green) in the normal, AP and duct ligation-induced SAP models (n=5). The fluorescence signal of Hspb1 was quantified using ImageJ software (upper), while the colocalization of Hspb1 and insulin in the AP group was also analyzed using ImageJ (lower). Scale bar, 50 μm. (K) IF staining of Hspb1 (green) in acinar cells (marked by amylase, red) from normal pancreas and pancreatic tissue samples from APs and chronic pancreatitis patients (n=4). The fluorescence signal of Hspb1 was quantified using ImageJ software (upper), while the colocalization of Hspb1 and amylase in the AP group was also analyzed using ImageJ (lower). Scale bar, 50 μm. Amy, amylase; ctrl, control; i.p., intraperitoneal injection; NC, negative control; ***p<0.001.

To understand the key contributors that promote the progression of AP to SAP, we used transcriptomics and proteomics to compare the gene and protein expression patterns among the pancreases of normal mice and mice with AP and SAP. AP was induced by 8 intraperitoneal injections of caerulein, and SAP was induced by pancreatic duct ligation followed by injection of caerulein (Fig. 1A). Transcriptomic analysis revealed 5820 and 1693 dysregulated genes according to a cutoff value of p<0.05 in the AP and SAP groups, respectively, compared to the control group (Fig. 1B, Fig. S1A-B). Hierarchical clustering analyses identified 4 major expression modules (Fig. S1C).

KEGG analysis of the differentially expressed genes between SAP and AP model mice revealed that several of the enriched signaling pathways are involved in programmed cell death (Fig. S1D). Additionally, a total of 6633 proteins across all of the samples were detected by proteomics (Fig. S1E-F). After the integration of transcriptomic and proteomic data, we found that 1400 and 802 DEGs existed between the AP vs. control groups and between the SAP vs. control groups (Fig. S1G). The reigning hypothesis is that there is a molecular mechanism within acinar cells that successfully limits the damage caused by AP, allowing the disease to resolve spontaneously without long-term consequences or progression to SAP; however, AP progresses when this mechanism collapses. To identify the key factors influencing the progression of AP to SAP, we screened the genes that were significantly upregulated in AP but attenuated in SAP and the genes and significantly downregulated in AP but upregulated in SAP, resulting in 25 and 18 candidates, respectively (Fig. 1C, Fig. S1H-I). Hspb1, an ATP-independent molecular chaperone, was previously reported to play a protective role in many diseases, but its biological effects and underlying mechanism in SAP are still unclear.

To verify the expression of Hspb1 in AP and SAP patients, we established two SAP models: a model induced by caerulein injections every 12 hours and a pancreatic duct ligation model. H&E staining of the pancreas revealed obvious acinar cell death and immune cell infiltration, and the plasma amylase and lipase levels were significantly higher in the model mice than in the control mice (Fig. 1D-E). Similar to the omics results, the RNA expression of Hspb1 was 4.17-fold higher than that in the control group but decreased 1.71-fold in the SAP group (Fig. 1F). Consistent with these findings, the protein expression of Hspb1 in the pancreas was higher in the AP group than in the normal group, whereas it was nearly decreased to the normal level in the SAP group (Fig. 1G). Furthermore, we investigated the major cell types that contributed to the upregulation of Hspb1 in AP. Insulin and Krt19 were used to mark islet and duct cells, respectively. Although it was notably upregulated in acinar cells, Hspb1 was not obviously changed in islet cells or duct cells in AP or SAP tissues compared to normal tissues (Fig. 1H-J). We further evaluated the expression of Hspb1 in patient samples and found that Hspb1 was obviously upregulated in AP samples compared to normal pancreas samples but was decreased in chronic pancreatitis (CP) samples, which are often the result of SAP (Fig. 1J). Thus, these data demonstrated that Hspb1 was upregulated in AP but attenuated in SAP, suggesting a potential role for Hspb1 in SAP.

### Inhibition or knockout of Hspb1 exacerbated AP, while intraductal administration of AAV8-Hspb1 significantly reduced SAP

**Figure 2:**
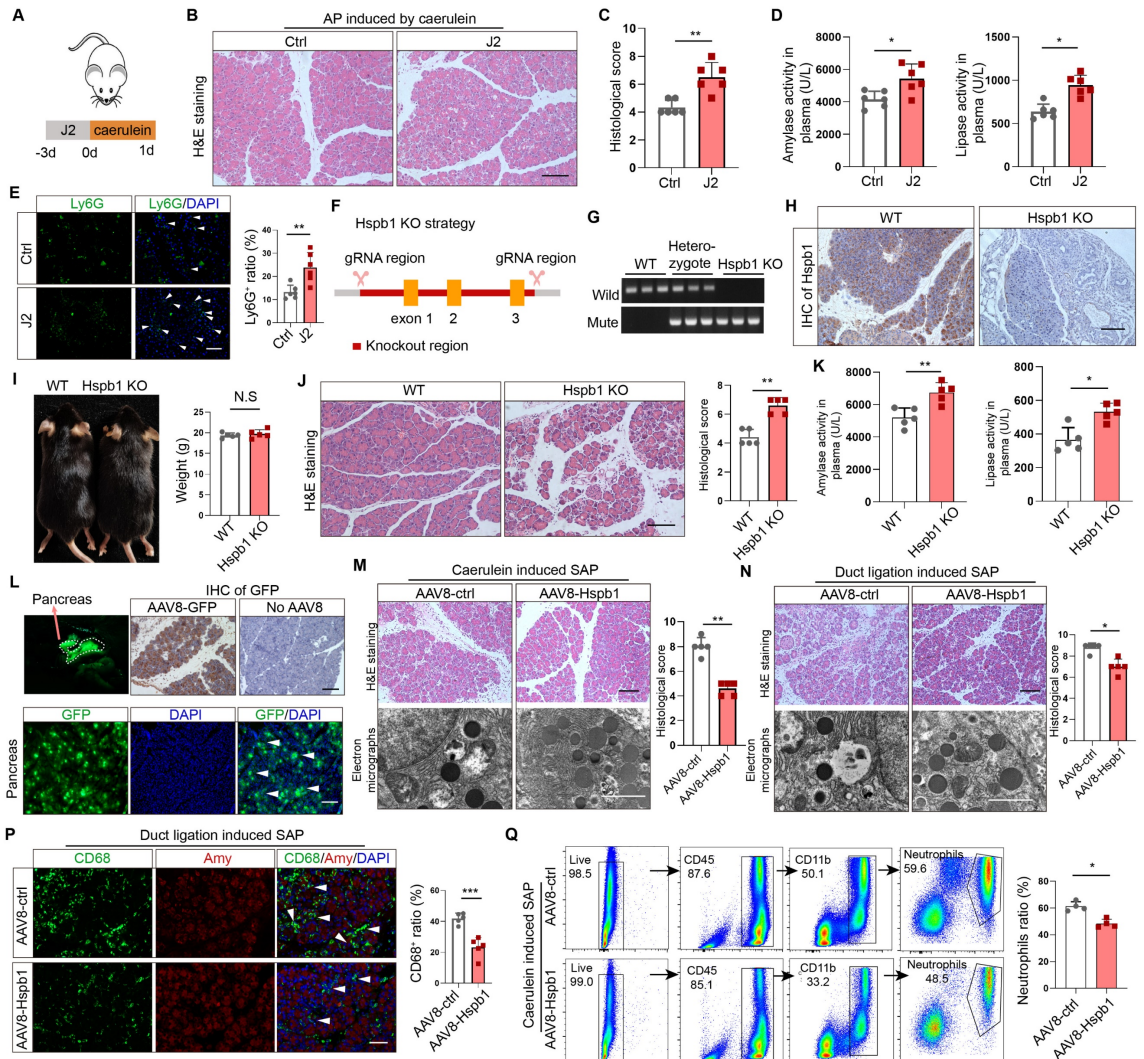
** Inhibition of Hspb1 worsened AP, while administration of Hspb1-AAV8 reduced SAP.** (A) J2 is a specific inhibitor of HSPB1. J2 (25 mg/kg) was intraperitoneally injected for three consecutive days, and then, 8 hourly injections of caerulein were used to induce AP. (B-D) H&E staining of the pancreas and amylase and lipase activity in plasma from the J2-treated group and the control group (n=6). The histological scoring of the pancreas was performed by a pathologist using the standards described in the methodology section. Scale bar, 100 μm. (E) Representative images and statistical analysis of Ly6G-positive cells in the pancreas of the J2-treated group and the control group (n=6). Scale bar, 50 μm. (F) Schematic diagram describing the strategy used to establish Hspb1-KO mice. (G) Genotypes of Hspb1 KO mice, heterozygous mice and WT mice determined by PCR and Southern blot analysis. For the mutated allele, the band size was 578 bp, and that for the wild-type allele was 546 bp. (H) Representative IHC image of Hspb1 in the pancreas of WT and Hspb1 KO mice. (I) At six weeks of age, the weights of the Hspb1 KO mice were comparable to those of the WT mice (n=5). (J-K) H&E staining of the pancreas and amylase and lipase activity in the plasma of the WT and Hspb1 KO mice (n=5). The histological scoring of the pancreas was performed by a pathologist using the standards described in the methodology section. Scale bar, 100 μm. (L) AAV8-GFP was used as a positive control. The pancreas was emitted a green fluorescence signal when it was irradiated using a handheld camera at a wavelength of 480 nm. Fresh pancreas tissue was cut into 4 μm-thick slices by a freezing microtome, and a strong GFP signal indicated successive expression of genes following intraduct administration of AAV8. Scale bar, 50 μm. (M-N) Representative image and statistical analysis of H&E staining of the pancreas and microstructural changes in the pancreas by scanning electron microscopy are shown for the AAV8-ctrl and AAV8-Hspb1 groups, which were generated by 12 hourly injections of caerulein or duct ligation, respectively (n=5). Scale bar, 100 μm for the top set and 2 μm for the bottom parts. (P) Representative images of immunofluorescence (IF) staining and statistical analysis of CD68 expression in the pancreas of the AAV8-ctrl and AAV8-Hspb1 groups, which were generated by duct ligation (n=5). Scale bar, 50 μm. (Q) Flow cytometry analysis of blood parameters of the AAV8-ctrl and AAV8-Hspb1 groups in the caerulein-induced SAP model (n=3). Ctrl, control. N.S., not significant; *p<0.05, **p<0.01.

To examine the role of Hspb1 in AP, we administered the Hspb1 inhibitor J2, which suppresses its chaperone function^28^, to mice and then injected them with caerulein to induce AP (Fig. 2A). H&E staining of pancreatic tissues showed enhanced edema and inflammatory cell infiltration induced by J2 (Fig. 2B-C). The plasma levels of amylase and lipase were also significantly increased (Fig. 2D). Ly6G was used to label neutrophils, and immunofluorescence staining confirmed that the number of infiltrating neutrophils was obviously increased by J2 (Fig. 2E). To further demonstrate the effect of Hspb1, we compared the severity of AP between Hspb1 KO and wild-type (WT) mice. The Hspb1 KO mice were constructed by CRISPR/Cas9, and the sgRNA was designed to target exons 1-3 (Fig. 2F). The genotype was identified by PCR and immunohistochemical staining of the pancreas (Fig. 2G-H). There was no significant difference in weight between the Hspb1 KO and WT mice (Fig. 2I). Like in J2 mice, Hspb1 KO obviously worsened pancreatic damage, elevated the level of pancreatic trypsin, and concurrently increased the infiltration of neutrophils in the pancreas (Fig. 2J-K, Fig. S2A). Thus, these data strongly showed that inhibiting or knocking out Hspb1 worsened AP by increasing pancreatic cell death and inflammatory cell infiltration.

A preliminary study showed that Hsbp1 overexpression could alleviate the severity of caerulein-induced AP^20^. To determine the role of Hspb1 in SAP, we administered adeno-associated virus 8 (AAV8) carrying Hspb1 (AAV8-Hspb1), via intraductal administration to both types of SAP model mice (Fig. S2B). An AAV8 vector carrying GFP was used as a positive control. Visualizing the pancreas, green fluorescence and immunofluorescence staining of GFP in tissue sections suggested the high efficiency of the AAV system in the pancreas (Fig. 2L). Fourteen days after the administration of AAV8, caerulein injections were administered every 12 hours to induce SAP. In the AAV8-Hspb1 group, the pancreases of the mice in the AAV8-Hspb1 group exhibited less edema, inflammatory cell infiltration, vacuolization, and necrosis than those of the control group (Fig. 2M). Electron micrographs showed a reduction in the number of deformed rough endoplasmic reticula (rERs), mitochondrial edema and deformed cristae in the AAV8-Hspb1 group compared to the control group (Fig. 2M). The average levels of amylase and lipase in plasma were lower than those in the control group, although the difference was marginally significant (p=0.0467 and 0.1137) (Fig. S2C). In the duct ligation model, specific overexpression of Hspb1 also reduced inflammatory cell infiltration, vacuolization, and necrosis in the pancreas and partially alleviated defects in the ER and mitochondria (Fig. 2N). Neutrophils and macrophages are the main inflammatory cells involved in AP. We determined the expression of Ly6G and CD68 (a marker of macrophages) in the pancreas. As expected, Hspb1 largely reduced the number of neutrophils and macrophages, although the basal number of these cells obviously differed among the different SAP models (Fig. 2P, Fig. S2D-E). Similarly, the number of neutrophils in the blood showed a similar trend (Fig. 2Q, Fig. S2F). Thus, these data clearly demonstrated that Hspb1 alleviates the severity of SAP in mice.

### Intraductal administration of AAV8-Hspb1 reduces lung and intestinal injury in SAP

**Figure 3:**
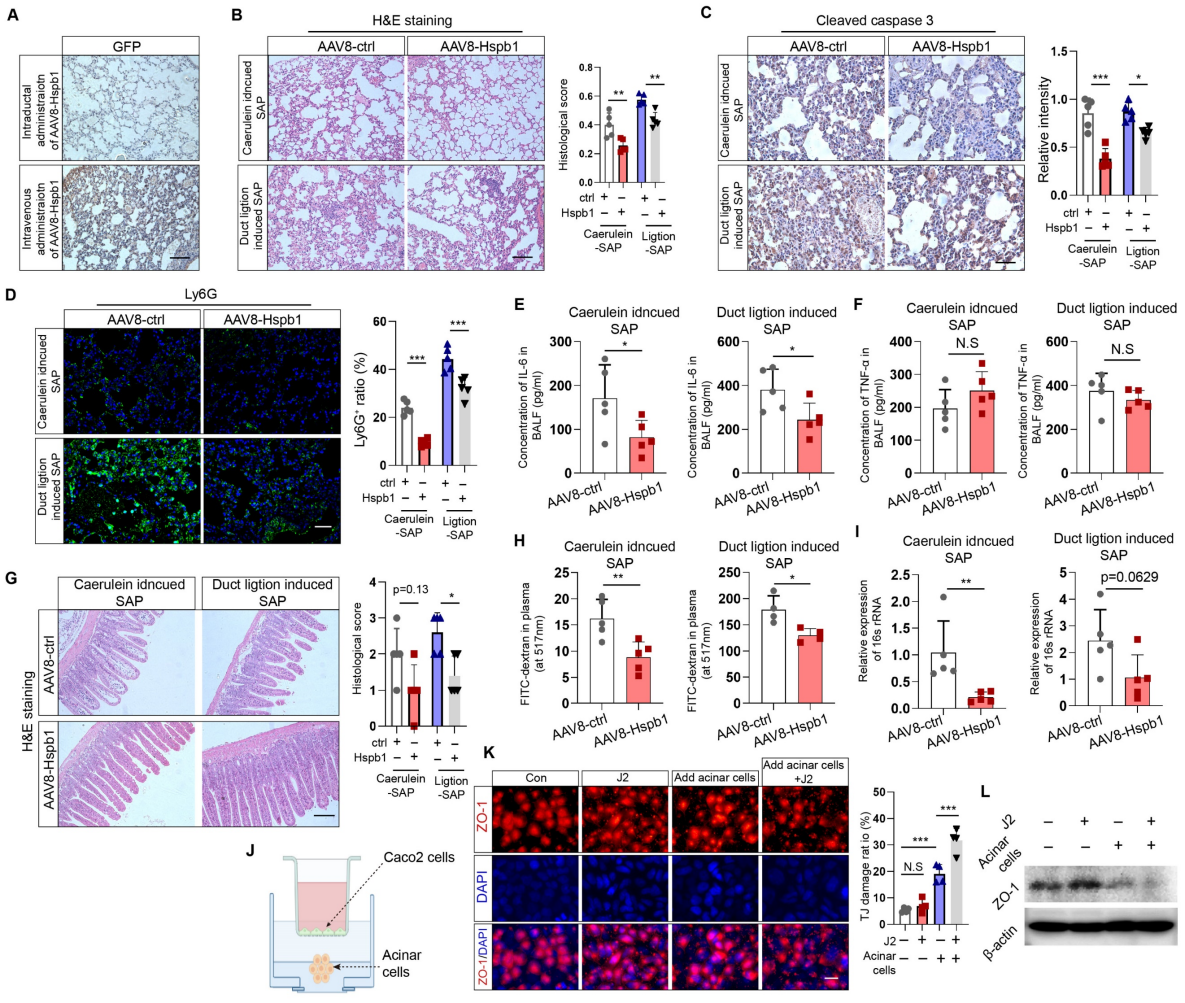
** AAV8-Hspb1 reduced lung and intestinal injury in SAP model mice.** (A) IHC staining of GFP in the lung after intraductal or intravenous injection of AAV8-GFP. Scale bar, 100 μm. (B) Representative image of H&E staining and statistical analysis of lung tissue from the AAV8-ctrl and AAV8-Hsbp1 groups of mice in the two SAP models (12 hourly injections of caerulein and duct ligation-induced model) (n=5). Scale bar, 100 μm. (C) IHC staining and statistical analysis of cleaved caspase 3 in the lungs of the AAV8-ctrl and AAV8-Hsbp1 mice in two SAP models (n=5). Scale bar, 100 μm. (D) Representative image of Ly6G expression and statistical analysis of lung tissue from the AAV8- ctrl and AAV8-Hsbp1 groups in two SAP models (n=5). Scale bar, 100 μm. (E-F) ELISAs of the IL-6 and TNF-α levels in BALF from the AAV8-con and AAV8-Hsbp1 groups of mice in two SAP models (n=5). (G) H&E staining and statistical analysis of intestinal tissue from the AAV8-ctrl and AAV8-Hsbp1 groups of mice in the two SAP models (n=5). The histological scoring of the intestine was performed by a pathologist using the standards described in the methodology section. Scale bar, 100 μm. (H) Gavage of FITC-dextran was used to evaluate gut permeability. The FITC signal in plasma was measured in the AAV8-ctrl and AAV8-Hsbp1 groups and is expressed as the relative fold change in the FITC signal. (n=5). (I) The expression of 16S rRNA in plasma was determined by qRT‒PCR. The relative RNA levels of 16S rRNA in the AAV8-ctrl and AAV8-Hsbp1 groups are presented (n=5). (J) Schematic of the coculture system of Caco-2 cells and primary acinar cells. To mitigate the potential impact of caerulein on Caco-2 cells, acinar cells were pretreated with caerulein, after which the supernatant containing caerulein was removed. (K) IF staining of ZO-1 in Caco-2 cells treated as indicated. Tight junctions (TJs) are specialized protein structures between adjacent epithelial cells. We quantified the number of cells exhibiting a loss of TJs. J2, 2.5 μM. (L) Western blot analysis of the expression of ZO-1 in Caco-2 cells treated as indicated. Ctrl, control; N.S., not significant; *p<0.05, **p<0.01.

Next, we investigated whether Hspb1 ameliorates SAP-induced damage to the lung and intestines. Acute lung injury is a major complication of SAP and is intimately associated with increased mortality. We examined the impact of Hspb1-AAV8 on SAP-associated lung injury and neutrophil infiltration. No GFP signal was detected in the lung, indicating that the potential impact of AAV8-Hspb1 on the lung was solely from Hspb1 in the pancreas (Fig. 3A). We found that AAV8-Hspb1 obviously alleviated the morphological changes in the lungs induced by SAP, including resumption of the alveolar septum, decreased alveolar edema and inflammatory cell infiltration in lung tissues (Fig. 3B). The expression of cleaved caspase 3 was attenuated, suggesting that apoptosis was decreased by AAV8-Hspb1 in SAP (Fig. 3C). The number of infiltrated neutrophils was also reduced in the lungs of the mice treated with AAV8-Hspb1 (Fig. 3D). To further examine the inflammatory response in the lung, we determined the levels of IL-6 and TNF-α in the bronchiolar alveolar lavage fluid (BALF). Despite no differences in TNF-α levels, a significant reduction in IL-6 levels was observed in the AAV8-Hspb1 group compared to the control group (Fig. 3E-F). Thus, these data suggested that Hspb1 could protect against lung injury during SAP.

Intestinal barrier dysfunction is also involved in the pathophysiology of SAP, especially SAP with septic complications. Therefore, we examined the impact of Hspb1 on the intestine and found a reduction in epithelial cell exfoliation and villus broadening in the AAV8-Hspb1 group compared to the control group in the two SAP models, especially in the pancreatic duct ligation models (Fig. 3G). Using a fluorescein isothiocyanate (FITC)-dextran-based gut permeability assay, we found that, compared with the control, AAV8-Hspb1 could decrease the leakage of intragastric FITC-dextran into the blood during SAP (Fig. 3H). Next, we detected the bacterial load in plasma by assessing the gene expression of 16S rRNA in SAP models. Interestingly, compared with the control, AAV8-Hspb1 inhibited the migration of gut bacteria into the blood, which might be associated with decreased intestinal integrity in SAP patients (Fig. 3I). Then, we asked whether acinar cells could directly damage intestinal epithelial cells in vitro. We used Caco-2 cells, a human colon adenocarcinoma cell line that is functionally similar to differentiated intestinal epithelial cells, in coculture with primary acinar cells. The coculture system is illustrated in Fig. 3J. We found that pretreatment of acinar cells with 100 nM caerulein decreased the expression of ZO-1 in Caco-2 cells, and inhibition of Hspb1 further reduced its expression (Fig. 3K-L). Thus, these data showed that the impact of Hspb1 from acinar cells on the intestinal barrier was, at least in part, derived from reducing gut permeability and bacterial translocation. In summary, Hspb1 in the pancreas could reduce lung and intestinal injury induced by SAP.

### Hspb1 inhibits the progression of AP into CP in a genetically engineered model

**Figure 4:**
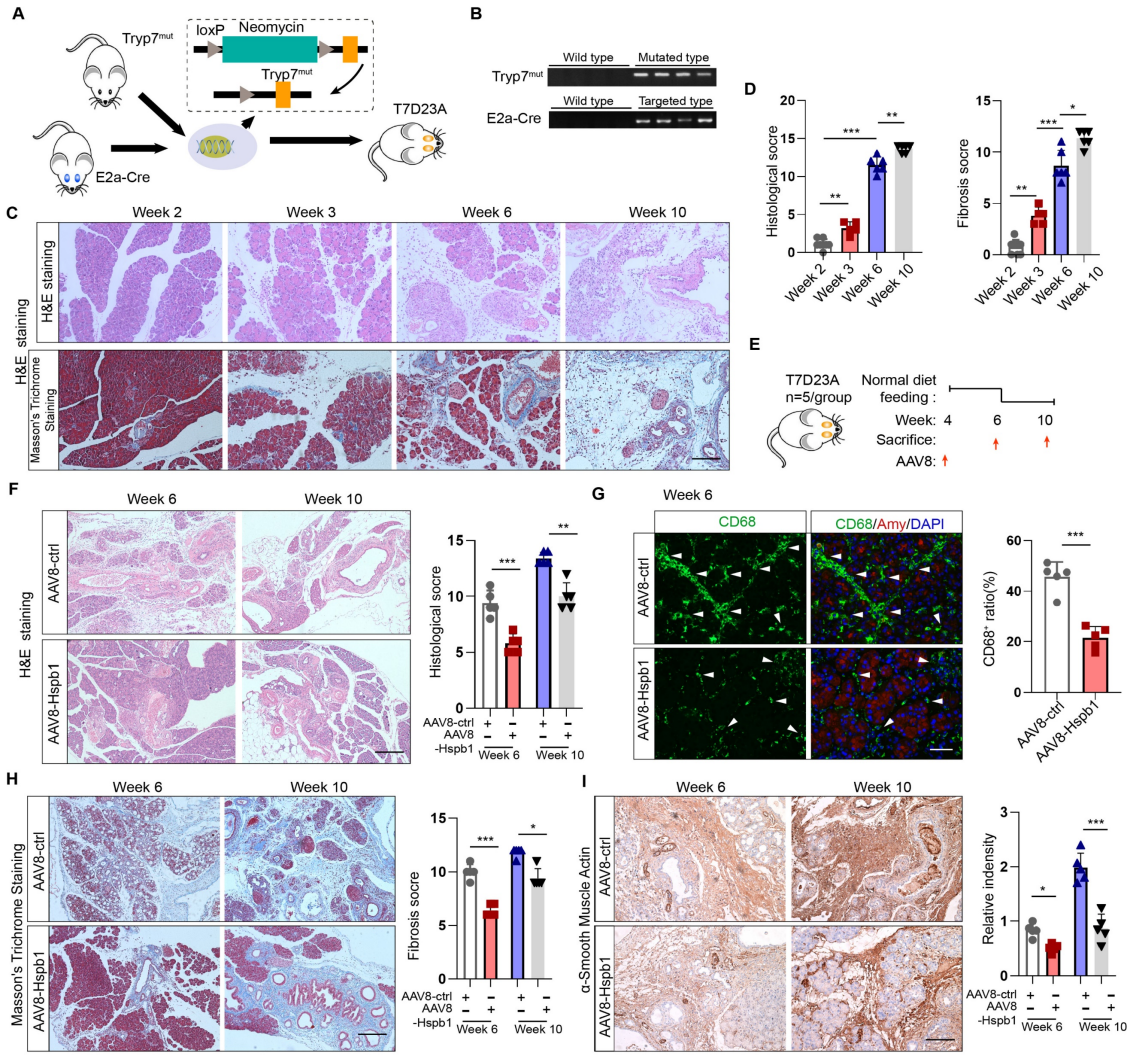
** Hspb1 inhibits the progression of AP into CP in a genetically engineered model.** (A) Schematic diagram describing the strategy used to establish the T7D23A strain. Tryp7^mut^ mice were crossed with E2a-Cre mice. (B) Southern blot image showing the genotype of the T7D23A mice. The band sizes were 537 bp for the mutated primer pair Tryp7^mut^ and 578 bp for the targeted primer pair E2a-Cre. (C-D) H&E staining, Masson's trichrome staining, and statistical analysis were performed to evaluate the pancreatic tissue of T7D23A mice aged 2, 3, 6, and 10 weeks (n=5-8). Scale bar, 100 μm. (E) Experimental design for T7D23A mice overexpressing AAV8-Hspb1. (F) H&E staining and statistical analysis of the pancreases of T7D23A mice aged 6 weeks and 10 weeks treated with AAV8-ctrl or AAV8-Hspb1 (n=5). Scale bar, 100 μm. (G) IF staining for CD68 was performed, and statistical analysis was also conducted to evaluate the effects of AAV8-ctrl or AAV8-Hspb1 treatment on 6-week-old T7D23A mice (n=5). Scale bar, 50 μm. (H) Masson's trichrome staining and statistical analysis were performed on the pancreases of 6- and 10-week-old T7D23A mice (n=5). Scale bar, 100 μm. (H) IHC staining and statistical analysis were conducted to evaluate the levels of αSMA in the pancreas of 6- and 10-week-old T7D23A mice treated with AAV8-ctrl or AAV8-Hspb1 (n=5). Scale bar, 100 μm. Amy, amylase; ctrl, control.

Since CP is the outcome of SAP, we wondered whether Hsbp1 affects this process. In accordance with previous methods^29^, a genetically engineered CP model was established by inserting the loxP-neomycin-loxP cassette and the p.D23A mutation (GAC to GCG) into exon 2 of the trypsinogen 7 precursor by homology-directed repair (named the Tryp7^mut^ strain). After crossing with E2a-Cre mice, trypsinogen 7 precursor mutant mice (named the T7D23A strain) were generated (Fig. 4A). The genotypes of these mice were verified by PCR (Fig. 4B). Consistent with the findings of previous reports, AP was observed at 3-6 weeks of age, early CP was observed at 5 weeks of age, and late CP was evident at 10 weeks of age, as shown by H&E and Masson's trichrome staining of the pancreas (Fig. 4C-D). To determine whether Hspb1 affects the process of AP development into the CP, we administered AAV8-Hspb1 at 4 weeks and collected the pancreas at 6 and 10 weeks (Fig. 4E). Strikingly, compared with those in the control group, the acinar cell damage in the AAV8-Hspb1 group was obviously reduced, and the number of surviving acini was increased at both 6 and 10 weeks (Fig. 4F). The number of infiltrated macrophages, which are essential for CP, was also obviously decreased by AAV8-Hspb1 (Fig. 4G). Furthermore, pancreatic fibrosis was reduced, as shown by Masson's trichrome staining and αSMA staining of the pancreas in the AAV8-Hspb1-treated mice (Fig. 4H-I). Thus, these results indicated that Hspb1 in the pancreas blocks AP from developing into CP likely by inhibiting acinar cell injury.

### Hspb1 reduced apoptosis and ferroptosis but not autophagy or necroptosis by eliminating intracellular ROS and lipid ROS in acinar cells

**Figure 5:**
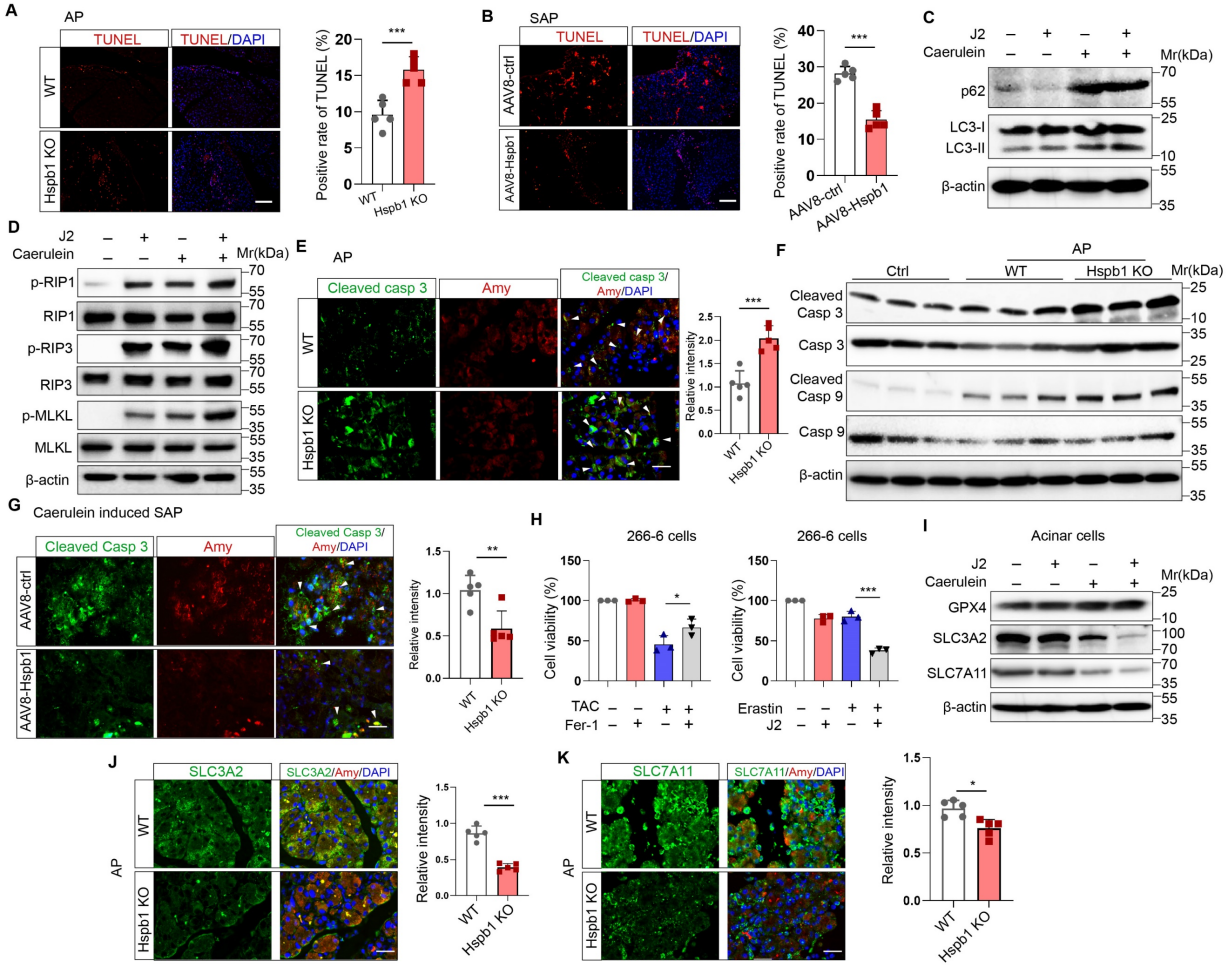
** Hspb1 reduced acinar cell apoptosis and ferroptosis but not autophagy or necroptosis.** (A) TUNEL assays of pancreatic samples from WT and Hspb1 KO mice treated with 8 hourly injections of caerulein (n=5). Scale bar, 50 μm. (B) TUNEL assay of pancreatic samples from the AAV8-ctrl and AAV8-Hspb1 groups treated with 12 hourly injections of caerulein (n=5). Scale bar, 50 μm. (C) Western blots of p62 and LC3 in primary acinar cells treated with 2.5 μM J2 or PBS and then stimulated with 100 nM caerulein for 6 hours. (D) Western blots of p-RIP1, RIP1, p-RIP3, RIP3, p-MLKL and MLKL in primary acinar cells treated with PBS or J2 followed by stimulation with 100 nM caerulein for 6 hours. (E) Representative images and statistical analysis of IF staining of cleaved caspase 3 (green) in acinar cells (marked by amylase, red) between the WT and Hspb1 KO mice with AP (n=5). Scale bar, 50 μm. (F) Western blots showing cleaved caspase 9 and cleaved caspase 3 in pancreatic tissue samples from WT and Hspb1 KO mice with AP. (G) Representative images and statistical analysis of IF staining of cleaved caspase 3 (green) in acinar cells (marked in red) from the AAV8-con and AAV8-Hspb1 SAP model mice(n=5). Scale bar, 50 μm. (H) Viability of 266-6 cells treated with 5 mM taurocholate acid in the presence or absence of 1 μM ferrostatin-1 (left). (A) Viability of 266-6 cells treated with 2.5 μM J2 in the presence or absence of 20 μM erastin-1 for 24 hours (right). n=3. (I) Protein expression of several determiners of ferroptosis, GPX4, SLC3A2, and SLC7A11, in primary acinar cells treated with 100 nM caerulein for 6 hours in the presence of 2.5 μM J2 or PBS. (J) Representative images and statistical analysis of SLC3A2 and SLC7A11 (green) expression in acinar cells (red) from WT and Hspb1-KO mice (n=5). Scale bar, 50 μm. (M) Representative images and statistical analysis of SLC3A2 and SLC7A11 (green) in acinar cells (red) from the AAV8-con and AAV8-Hspb1 mice in the SAP model (n=5). Scale bar, 50 μm. Casp, caspase; Amy, amylase; TAC, taurocholate acid; Fer, ferrostatin-1; *p<0.05, ***p<0.001.

After demonstrating the protective effect of Hspb1 on SAP, we explored the underlying mechanism. TUNEL assays showed that Hspb1 KO significantly increased the number of dead cells in the AP model, while AAV8-Hspb1 largely rescued cell death in the SAP model (Fig. 5A-B). In isolated acinar cells, 2.5 μM J2 did not affect the expression of the autophagy markers LC3 and p62 upon treatment with 100 nM caerulein (Figure 5C). The RIPK1/RIPK3/MLKL1 axis mediates necroptosis, and no changes in the expression of these proteins were affected by J2 in acinar cells (Fig. 5D). In contrast, the expression of the apoptosis effectors cleaved caspase 3 and cleaved caspase 9 was upregulated in Hspb1 KO mice (Fig. 5E-F, Fig. S3A-B). Moreover, AAV8-Hspb1 rescued apoptosis, as indicated by decreased staining of cleaved caspase 3 in the pancreas in SAP model mice (Fig. 5G, Fig. S3C-D). On the other hand, treatment with an inhibitor of ferroptosis, ferrostatin-1 (Fer-1), increased the viability of 266-6 cells upon treatment with 5 mM taurocholate acid (Fig. 5H), confirming that ferroptosis participates in the process of acinar cell death. Additionally, J2 decreased the viability of 266-6 cells when they were cotreated with erastin, an activator of ferroptosis (Fig. 5H). Then, we determined the expression of ferroptosis-associated proteins in vitro and in vivo. In caerulein-treated primary acinar cells, GPX4 expression was not changed by J2, but SLC3A2 and SLC7A11 were obviously downregulated (Fig. 5I). Consistent with these findings, the Hspb1 KO mice exhibited lower SLC3A2 and SLC7A11 expression, while GPX4 expression was not changed in the caerulein-induced AP model (Fig. 5J-K, Fig. S3E-G). Thus, these data suggested that Hspb1 reduced acinar cell apoptosis and ferroptosis but not autophagy or necroptosis.

**Figure 6:**
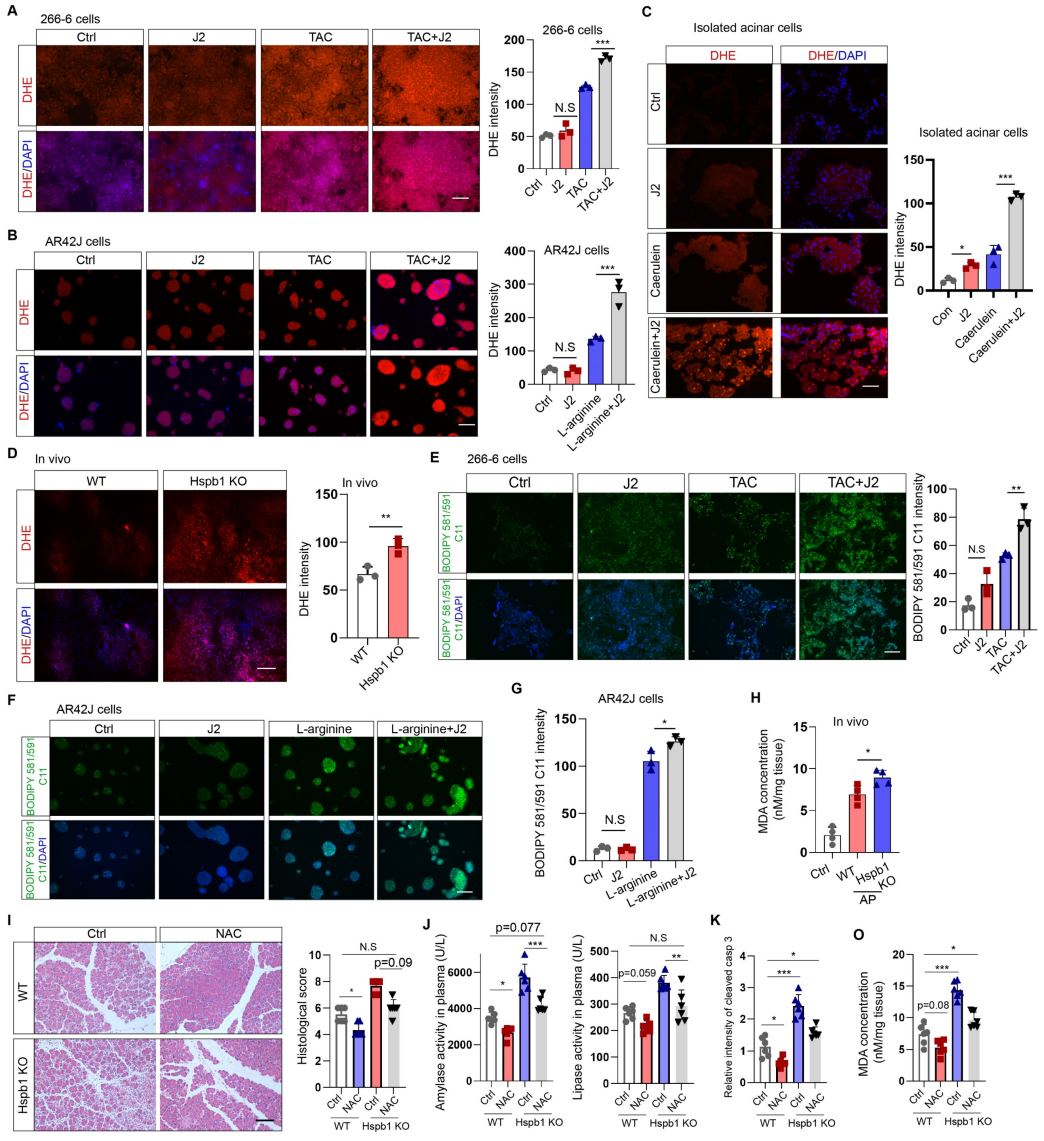
**Hspb1 attenuated intracellular ROS and lipid ROS levels.** (A) 266-6 cells were pretreated with 2.5 μM J2 or PBS for 2 hours and then treated with 5 mM taurocholate acid for 24 hours. Representative images of the fluorescence signal at a wavelength of 594 nm after incubation with 5 μM DHE for 10 min. Quantitative analysis of the signal from the indicated groups. Scale bar, 50 μm. n=3. (B) Representative images and quantitative results for AR42J cells. L-arginine at 10 mg/ml was added for 24 hours before the cells were incubated with 5 μM DHE for 10 min. Scale bar, 50 μm. n=3. (C) Representative images and quantitative results for isolated acinar cells. Caerulein (100 nM) was added before the cells were incubated with 5 μM DHE for 10 min. Scale bar, 50 μm. n=3. (D) A total of 200 μl of 50 μM DHE was infused into the pancreas, which was subsequently incubated for 30 min. Representative images of the pancreases of the WT and Hspb1 KO mice with AP are shown. Scale bar, 50 μm. n=3. (E) 266-6 cells were pretreated with 2.5 μM J2 or PBS for 2 hours and then treated with 5 mM taurocholate acid for 24 hours. Representative images of the fluorescence signal at 488 nm after incubation with 2.5 μM BODIPY 581/591 C11 for 30 min. Scale bar, 50 μm. (F-G) Representative images and quantitative results for AR42J cells treated as indicated. Scale bar, 50 μm. n=3. (H) Comparison of the MDA levels in the pancreas between WT and Hspb1 KO mice with AP (n=4). (I) H&E staining and statistical analysis were performed to evaluate pancreatic tissue from WT and Hspb1 KO mice treated as indicated. NAC was administered at a dose of 150 mg/kg body weight prior to the initial caerulein injection (n=6). (J) Amylase and lipase activity in plasma was assessed in both WT and Hspb1 KO mice, with or without NAC treatment (n=6). (K) The expression of cleaved caspase 3 in the pancreas was assessed using IHC staining, followed by statistical analysis (n=6). (O) The MDA levels in the pancreas were compared between WT and Hspb1 KO mice, both with and without NAC treatment, in the context of AP (n=6). Ctrl, control; TAC, taurocholate acid; N.S., not significant; *p<0.05, **p<0.01, ***p<0.001.

Hspb1 was previously found to be essential for protecting against cellular oxidative stress. To evaluate cellular ROS levels, we incubated DHE (dihydroethidium, a cell-permeable fluorescent probe) with 266-6 mouse acinar cells, AR42J rat acinar cells, and primary acinar cells. J2 did not promote obvious ROS production in 266-6 or AR42J cells in the absence of stimulating factors (Fig. 6A-B). The same concentration of J2 resulted in increased ROS production in primary acinar cells, which might be explained by the fragility of freshly isolated acinar cells (Fig. 6C). In contrast, J2 significantly increased cellular ROS levels in 266-6 cells upon stimulation with 5 mM taurocholate acid, in AR42J cells stimulated with 10 mg/ml L-arginine and in primary acinar cells stimulated with 100 nM caerulein (Fig. 6A-C). Next, we examined whether Hspb1 KO could increase cellular ROS levels in vivo. Twenty-four hours after the first injection of caerulein, the mice were dissected via a midline abdominal incision, and 200 µl of 50 μM DHE was infused into the pancreatic duct. Then, the pancreas was excised, cut into 4 μm-thick slices, and excited by a 488 nm wavelength light. The results indicated that knockout of Hspb1 promoted cellular ROS production in acinar cells during SAP (Fig. 6D).

As ferroptosis is driven by Fe^2+^-dependent lipid oxidation, we measured whether Hspb1 affects the level of lipid ROS via BODIPY 581/591 C11. Under basal conditions, nearly no signal was detected by fluorescence microscopy with BODIPY 581/591 C11. In contrast, the stimulating factors induced an intense green signal in 266-6 and AR42J cells, which was further increased by J2 (Fig. 6E-G). To further clarify the role of Hspb1 in regulating ferroptosis, we used a malondialdehyde (MDA) assay to assess lipid peroxidation in tissues. Among the Hspb1 KO mice, the pancreas contained more MDA than the pancreas of the WT mice treated with caerulein (Fig. 6H). To further clarify whether Hspb1 controls acinar cell apoptosis and ferroptosis in a ROS-dependent manner, we administered N-acetylcysteine (NAC), an ROS scavenger, to WT and Hspb1 KO mice and induced AP. The results showed that NAC administration reduced pancreatic pathological damage and the levels of plasma amylase and lipase in WT mice, which is consistent with the findings of previous research^30^ (Fig. 6I-J). Interestingly, NAC treatment significantly decreased pancreatic pathological damage and plasma amylase and lipase levels in Hspb1 KO mice and restored these levels nearly to the levels observed in WT mice (Fig. 6I-J). Furthermore, we found that the administration of NAC resulted in a reduction in pancreatic apoptosis, as indicated by cleaved caspase 3 staining, and decreased ferroptosis, as measured by MDA levels (Fig. 6K-O). Thus, these data demonstrated that Hspb1 is essential for defending against oxidative stress in acinar cells during SAP.

### Hspb1 interacts with Anxa2 to control its aggregation and phosphorylation

**Figure 7:**
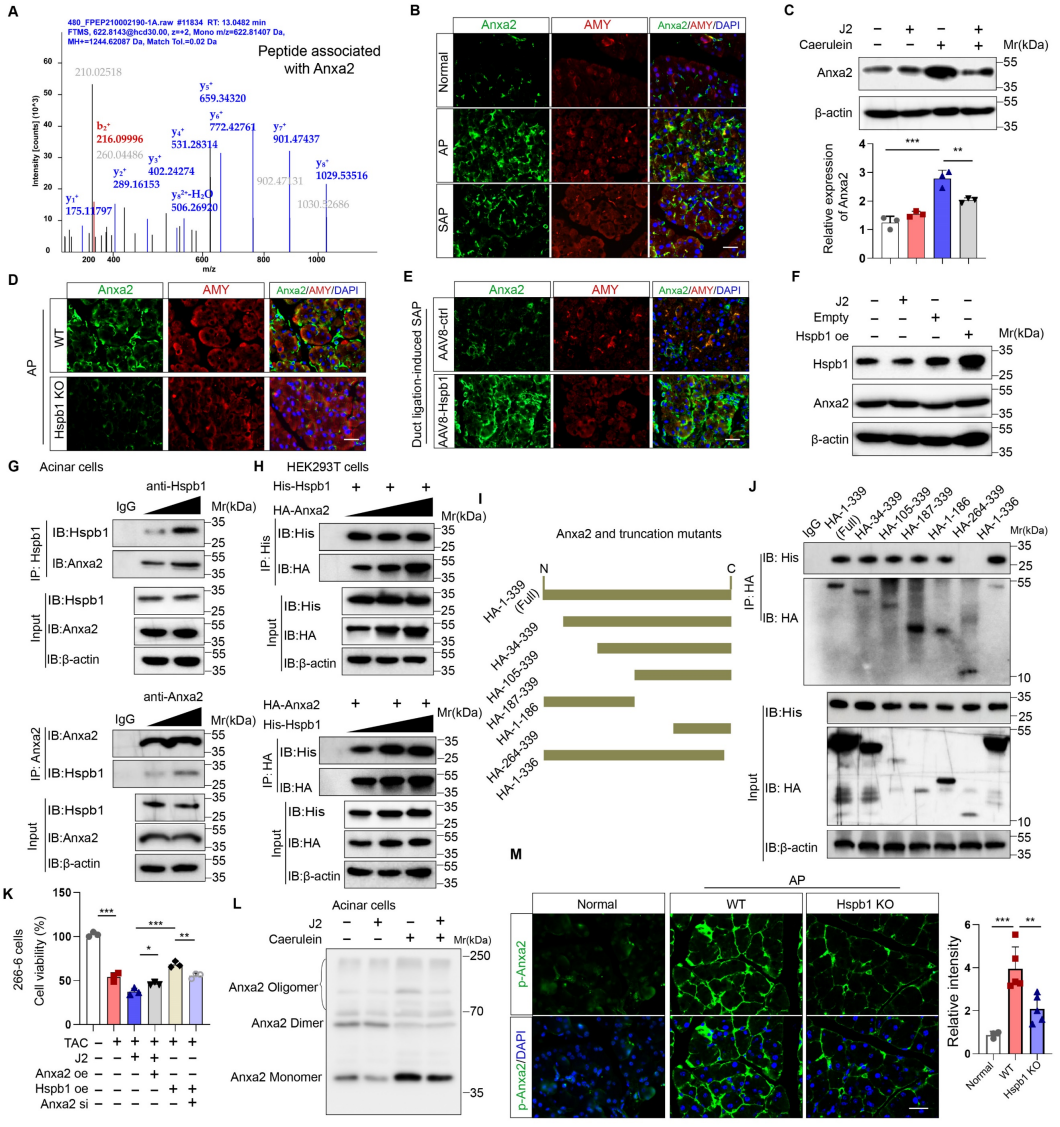
** Hspb1 interacted with Anxa2 and controlled its aggregation and phosphorylation.** (A) Hspb1 was enriched in primary acinar cells treated with 100 nM caerulein for 6 hours by an anti-Hspb1 antibody, and the binding peptides were analyzed via mass spectrometry. The peptide belonging to Anxa2 was extracted. (B) Representative images of Anxa2 (green) expressed in acinar cells (red) in the normal, AP and SAP groups. Scale bar, 50 μm. (C) Protein expression of Anxa2 in acinar cells pretreated with 2.5 μM J2 or PBS for 2 hours and then treated with 100 nM caerulein for 6 hours. n=3. (D-E) Representative images of Anxa2 (green) in acinar cells (red) from the WT and Hspb1 KO mice with AP (left) and between the AAV8-ctrl and AAV8-Hspb1 groups with SAP (right). Scale bar, 50 μm. (F) A plasmid harboring Hspb1 was transfected into 266-6 cells for 48 hours, and 2.5 μM J2 was added for 24 hours. The protein expression of Anxa2 is presented for the indicated groups. (G) Anti-Hspb1 or anti-Anxa2 antibodies were used to enrich associated proteins in primary acinar cells, and the precipitate was subjected to Western blotting. (H) In HEK293T cells, plasmids were transfected as indicated. His was fused to the N-terminus of the Hspb1 protein, and HA was fused to the N-terminus of the Anxa2 protein. An anti-His antibody and an anti-HA antibody were used for the immunoprecipitation assay. The expression of His and HA was detected via Western blotting. (I) A series of HA-Anxa2 truncation mutants were generated as indicated. (J) Plasmids were transfected into HEK293T cells in the indicated groups for 48 hours. An anti-HA antibody was used for the IP assay, after which the expression of His was detected. (K) Transfection of 266-6 cells with Anxa2, Hspb1 or Anxa2 siRNA was performed as indicated, and the cells were incubated for 48 hours and then incubated with 5 mM taurocholate acid for 24 hours. CCK-8 assays were used to determine cell viability (n=3). (L) Blue-native PAGE showed a lower proportion of monomers in the control group than in the J2 treatment group among the isolated acinar cells. (M) Representative images of p-ANXA2-expressing cells in the pancreas of WT and Hspb1-KO mice with AP. Scale bar, 50 μm. Ctrl, control; N.S., not significant; **p<0.01, ***p<0.001.

To explore the molecular mechanism by which Hspb1 protects against SAP, we used mass spectrometry to analyze the pulldown proteins associated with Hspb1 in primary acinar cells treated with 100 nM caerulein. A total of 78 potential interactors were identified, and we focused on Anxa2, a key annexin essential for injury and redox control (Fig. 7A). Anxa2 was markedly upregulated in AP but attenuated in SAP (Fig. 7B), which was consistent with the expression pattern of Hspb1. We subsequently examined whether Hspb1 affects the expression of the Anxa2 protein. Pretreatment with J2 did not change the expression of Anxa2 in primary acinar cells under basal conditions but did decrease its expression in the presence of 100 nM caerulein (Fig. 7C). Consistent with these findings, the Hspb1 KO mice presented slightly lower expression of Anxa2 in the pancreas than the control mice, and conversely, the expression of AAV8-Hspb1 was increased (Fig. 7D-E, Fig. S4A-D). To determine whether Hspb1 directly regulates the expression of Anxa2 in acinar cells, we overexpressed or inhibited Hspb1 in 266-6 cells, and the results showed no significant difference among the groups (Fig. 7F), indicating that Hspb1 did not directly affect the expression of Anxa2. Thus, the difference in Anxa2 expression between the WT and Hspb1 KO, AAV8-ctrl and AAV8-Hspb1 groups might be determined by the severity of AP.

Next, we treated isolated primary acinar cells with 100 nM caerulein and incubated them with antibodies against Hspb1 and Anxa2 for immunoprecipitation assays. Consistent with the abovementioned results, Hspb1 precipitated Anxa2 in a dose-dependent manner, and accordingly, Anxa2 also precipitated Hspb1 (Fig. 7G). In HEK293T cells, we cotransfected His-tagged Hspb1 (His-Hspb1) and HA-tagged Anxa2 (HA-Anxa2) plasmids for 48 hours and then used His- and HA-tagged antibodies for the immunoprecipitation assay. As expected, the His antibody precipitated HA, and conversely, the HA antibody also precipitated His in a dose-dependent manner (Figure 7H). To determine the interaction of the Anxa2 region with Hspb1, we generated a series of HA-tagged Anxa2 truncation mutants and separately transfected them with His-Hspb1 in HEK293T cells (Fig. 7I). Most of the HA-Anxa2 and HA-Anxa2 truncation mutants markedly precipitated His-Hspb1, except for HA-264-339, suggesting that the protein region 105-263 was essential for the binding of Anxa2 to Hspb1 (Fig. 7J).

As we demonstrated the interaction of Hspb1 with Anxa2, we next explored the Anxa2-mediated functional effects of Hspb1. In 266-6 cells, overexpression of Anxa2 rescued the inhibitory effect of J2 after treatment with 5 mM taurocholate acid, whereas knockdown of Anxa2 largely blocked the protective effect of Hspb1 overexpression (Fig. 7K), suggesting that the function of Hspb1 was largely determined by Anxa2. To demonstrate that Anxa2 is a key downstream mediator of Hspb1 function in vivo, we performed pancreatic ductal infusion of AAV8-Hspb1 in Anxa2 KO mice followed by intraperitoneal injection of caerulein to induce SAP (Fig. S5A). The results showed that the severity of AP was exacerbated in Anxa2 KO mice (Fig. S5B-D). While overexpression of Hspb1 decreased plasma amylase levels, there were no significant changes observed in tissue histopathology or plasma lipase expression (Fig. S5B-D). Thus, our data clearly demonstrated that Anxa2 plays a crucial role in the function of Hspb1 in acinar cells.

Hspb1 is a family of molecular chaperones that maintain protein solubility and limit protein aggregation. Thus, we used blue-native PAGE to explore whether J2 influences the aggregation state of Anxa2, and the results showed that J2 decreased the proportion of monomers in 266-6 cells (Fig. 7L). In addition, Tyr-23 phosphorylation of Anxa2 correlates with actin remodeling, a protective event against oxidative stress^31^. In the Hspb1 KO mice, the Tyr-23 phosphorylation level of Anxa2 was lower than that in the WT mice (Fig. 7M, Fig. S4E-F). Next, we investigated whether Anxa2 was essential for Hspb1-mediated antioxidative activity in acinar cells. After treatment with 5 mM taurocholate acid, Anxa2 knockdown largely promoted the levels of ROS and lipid ROS in 266-6 cells overexpressing Hspb1 (Fig. S5E-F). Previous works suggested that Tyr-23 phosphorylation and aggregation of Anxa2 influence the redox balance and interactions with other proteins, leading to functional changes and potential implications for diseases^32^, ^33^. Therefore, these data demonstrated that Hspb1 interacts with Anxa2 to control its aggregation and phosphorylation and combat oxidative stress in acinar cells.

### Hspb1 cooperates with Anxa2 to maintain the antioxidative activity of Prdx1

**Figure 8:**
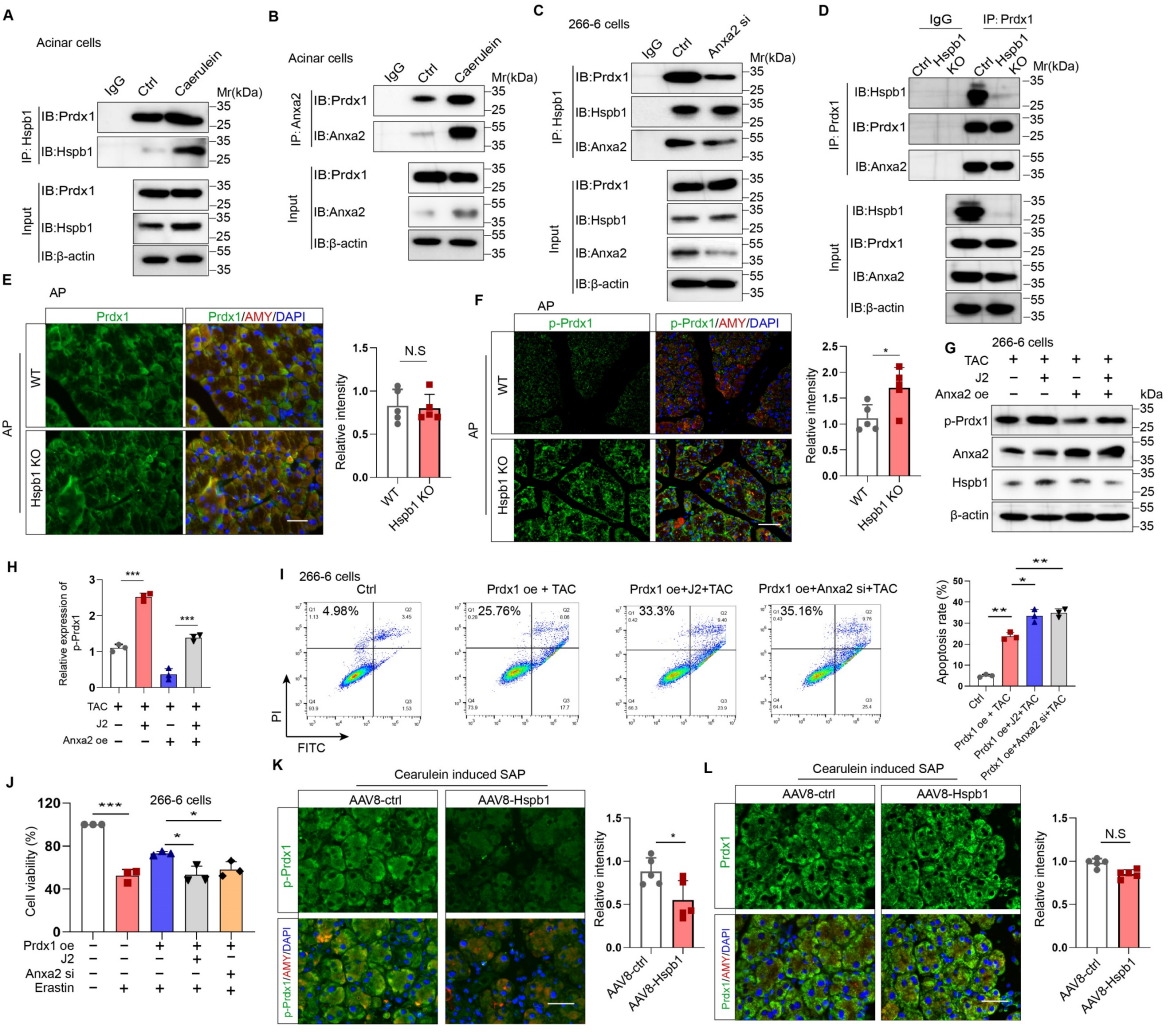
**Hspb1 cooperated with Anxa2 to maintain the antioxidative activity of Prdx1.** (A-B) Primary acinar cells were treated with 100 nM caerulein or PBS for 6 hours, and anti-Hspb1 or anti-Anxa2 antibodies were used for IP assays. The expression of Hspb1, Anxa2 and Prdx1 in the input and IP pools is shown. (C) Expression of Hspb1, Anxa2 and Prdx1 in the input and IP pools determined by an anti-Hspb1 antibody in the ctrl or Anxa2 siRNA groups. (D) The expression levels of Hspb1, Anxa2, and Prdx1 were assessed in the input and IP pools using an anti-Prdx1 antibody in both ctrl and Hspb1 KO cells derived from the 266-6 cell line. (E-F) Representative images and statistical analysis of Prdx1 or p-Prdx1 (green) expression in acinar cells (red) from WT and Hspb1-KO mice with AP (n=5). Scale bar, 50 μm. (G-H) Expression of p-Prdx1 in 266-6 cells treated as indicated. (I) Flow cytometry analysis of the apoptosis rate of 266-6 cells treated as indicated. (J) The viability of the 266-6 cells in the indicated groups was detected by a CCK-8 assay (n=3). (K-L) Representative images and statistical analysis of Prdx1 and p-Prdx1 (green) expression in acinar cells (red) in the AAV8-control and AAV8-Hsbp1 groups established by 12 hourly injections of caerulein (n=5). Scale bar, 50 μm. Ctrl, control; TAC, taurocholate acid; oe, overexpression; si, siRNA. *p<0.5, **p<0.01, ***p<0.001.

We then explored the precise mechanisms by which Hspb1 alleviates oxidative stress though Anxa2. Previous works have suggested that Anxa2 often serves as a scaffolding protein for template molecular interactions. Among the proteins in the Hspb1 precipitate, Prdx1, which belongs to the peroxiredoxin family of antioxidant enzymes, is related to a stress signaling kinase. We confirmed that Hspb1 could bind to Prdx1 under basal conditions, and strong binding was detected upon stimulation of isolated acinar cells with 100 nM caerulein (Fig. 8A). Furthermore, in the pool precipitated with the anti-Anxa2 antibody, we also detected enrichment of the Prdx1 protein (Fig. 8B). Next, we investigated whether Anxa2 was the key bridge for the interaction between Hspb1 and Prdx1 in acinar cells. In 266-6 cells, knockdown of Anxa2 largely abrogated the binding of Hspb1 to Prdx1 (Fig. 8C), indicating that Hspb1 interacted with Anxa2 to bind with Prdx1. To provide compelling evidence regarding the critical role of Hspb1 in mediating the interaction between Anxa2 and Prdx1, we first generated a Hspb1 KO cell based on the 266-6 cell line. Subsequently, we compared the binding ability of Anxa2 and Prdx1 in these and control cells. The results demonstrated a reduction in the binding of Anxa2 and Prdx1 within the context of Hsbp1 KO in the 266-6 cells (Fig. 8D). This finding conclusively confirms that the interaction between Anxa2 and Prdx1 is indeed influenced by Hspb1.

Next, we explored the functional effect of Anxa2 on Prdx1 in acinar cells. Similarly, compared with that in the WT mice, the protein expression of Prdx1 in the pancreas was not significantly changed in the Hspb1 KO mice, while its phosphorylation at Thr90 was obviously increased (Fig. 8E-F, Fig. S6A-C). Previous works have shown that the phosphorylation of Thr-90 attenuates the antioxidation function of Prdx1^34^. Thus, we hypothesized that Anxa2 could interact with Prdx1 to regulate its phosphorylation at Thr90. In the presence of taurocholate acid, overexpression of Anxa2 decreased the phosphorylation of Prdx1 in 266-6 cells, and interestingly, J2 abrogated this effect (Fig. 8G). Functionally, inhibition of Hspb1 by J2 or knockdown of Anxa2 led to increased apoptosis and ferroptosis in 266-6 cells overexpressing Prdx1 (Fig. 8H-J). Furthermore, AAV8-Hspb1 markedly decreased the phosphorylation of Thr-90 of Prdx1 in acinar cells during SAP, although no change in Prdx1 was detected between the AAV8-Hspb1 and control groups (Fig. 8K-L, Fig. S6D-F). Thus, these data suggested that Hspb1 synergizes with Anxa2 to maintain the enzymatic activity of Prdx1, inhibiting apoptosis and ferroptosis during SAP.

### Hspb1 protects against SAP in a Prdx1-dependent manner

**Figure 9:**
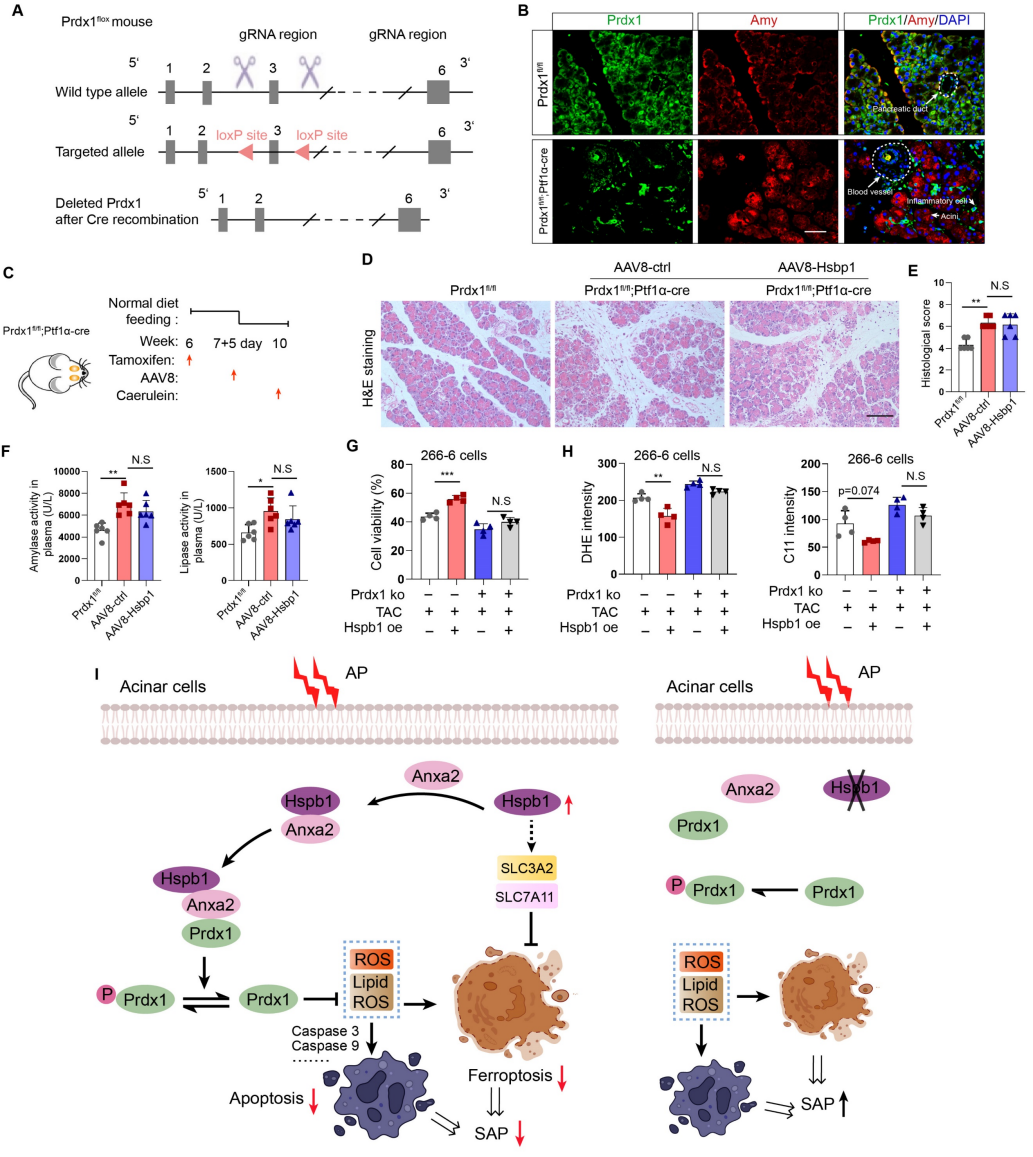
** Hspb1 protects against SAP in a Prdx1-dependent manner.** (A) Schematic diagram describing the strategy used to establish Prdx1-cKO mice. Prdx1^flox^ mice were crossed with Ptf1a-Cre mice, and acinar-specific knockout of Prdx1 was induced by tamoxifen injection. (B) Representative IF staining results showing the efficiency and specificity of acinar cell-specific Prdx1 deletion in Prdx1-cKO mice. Scale bar, 50 μm. (C) Experimental design for Prdx1-cKO mice overexpressing AAV8-Hspb1 or AAV8-ctrl. (D) H&E staining of pancreatic tissues from the various groups, as indicated. Scale bar, 100 μm. (E) Histological scores of the pancreas in the various groups, as indicated. (F) Amylase and lipase activity in plasma from the various groups, as indicated. (G) Prdx1 was knocked out in 266-6 cells using CRISPR‒Cas9. The results showed the viability of 266-6 wild-type and 266-6 Prdx1 knockout cells treated with TAC. (H) Quantitative analysis of the signals from the indicated groups. (I) A proposed working model of Hspb1-mediated protection against SAP. Ctrl, control; TAC, taurocholate acid; KO, knockout; fl, flox; N.S, not significant; *p<0.5, **p<0.01, ***p<0.001.

To demonstrate that Prdx1 is the key downstream target of Hspb1 in SAP, we evaluated the effects of AAV8-Hspb1 on an acinar-specific Prdx1 knockout mouse strain (Prdx1-cKO). The targeting strategy for Prdx1^fl/fl^ mice is shown in Fig. 9A. After crossing with Ptf1α-Cre (in which Cre recombinase was expressed in acinar cells), Prdx1-cKO was established, and the knockout efficiency and specificity in acinar cells were demonstrated by IF staining (Fig. 9B). AAV8-Hspb1 or AAV8-ctrl was intraductally administered 7 days after intraperitoneal injection of tamoxifen, and AP was induced by 8 hours of caerulein injection two weeks after AAV8 administration (Fig. 9C). As shown by H&E staining of the pancreas, compared with Prdx1fl/fl mice, Prdx1-cKO mice exhibited greater infiltration of inflammatory cells, vacuolization, and cell death (Fig. 9D-E). Additionally, the activity of amylase and lipase in the plasma increased in Prdx1-cKO mice (Fig. 9F). Strikingly, the overexpression of Hspb1 did not reduce the degree of histological damage or the activity of amylase or lipase in the plasma of Prdx1-cKO mice (Fig. 9D-F). Furthermore, overexpression of Hsbp1 did not rescue cell death in Prdx1 knockout cells (Fig. 9G). No differences in cellular ROS or lipid ROS were detected between the Hsbp1 overexpression group and the control group when Prdx1 was knocked out (Fig. 9H). Thus, these data suggested that Hspb1 protects against SAP through a Prdx1-dependent pathway.

## Discussion

SAP is a difficult-to-treat disease with a high mortality rate that is caused by systematic injury and multiple-organ failure. Decades of studies have resulted in major progress in understanding the pathological processes and potential mediators of systematic injury that occur during SAP. These mediators include trypsin, neutrophils, damage-associated molecular patterns (DAMPs), inflammasomes, and the coagulation system^35^-^38^. However, clinical pharmacological targets for treating SAP are largely supportive, and specific therapies are still unsatisfactory^25^. AP originates in acinar cells, where pancreatic trypsinogen is preactivated by pathogenic factors. In response to acinar injury, a pronounced protective action is initiated, and thus, most AP patients show limited symptoms^25^. However, when the balance between the protective response and injury is disrupted or the parallel systematic inflammatory response is not controlled, the disease progresses to SAP^39^. In our study, we screened for variations in gene expression among normal pancreatic, AP and SAP tissue samples from mice via transcriptomics and proteomics and found that Hspb1 was highly upregulated in AP but attenuated in SAP. Importantly, knockout or inhibition of Hspb1 worsened AP, while pancreatic duct administration of AAV8-Hspb1 alleviated the severity of SAP in two SAP models. Hspb1 reduced apoptosis and ferroptosis but did not affect autophagy or necroptosis, two other types of programmed cell death in acinar cells. We further demonstrated that Hspb1 interacted with Anxa2 to decrease Prdx1 phosphorylation and maintain its antioxidative activity in acinar cells. Our results suggest that the Hspb1/Anxa2/Prdx1 pathway is an essential protective signaling pathway for AP and may provide an alternative therapeutic strategy for SAP (as illustrated in Fig. 9I).

At the onset of SAP, the inflammatory response is determined by the severity of acinar cell injury, followed by secondary infection, which contributes mainly to bacterial translocation from the intestine^40^. For instance, high-mobility group box 1 (HMGB1), which is a type of DAMP, is positively correlated with AP severity in humans^41^-^43^.

HMGB1 can activate inflammasomes in macrophages and neutrophils by binding to Toll-like receptors and thus worsen AP^44^. Histones expelled from damaged acinar cells promote the inflammatory response by inducing pyroptosis and necroptosis in immune cells^45^. Interestingly, several works have suggested that HSPs expelled by necrosis can also serve as DAMPs recognized by Toll-like receptors, thereby inducing inflammation 46. In contrast, our work, together with previous reports, showed that the inhibition of intracellular Hspb1 by pharmacological or genetic methods worsens AP, but the overexpression of Hspb1 largely protects against SAP by inhibiting apoptosis and ferroptosis in acinar cells. We also found that intracellular Hspb1 overexpression can reduce the infiltration of inflammatory cells into the pancreas and remote organs. In addition, previous reports suggested that Hspb1 can inhibit the release of DAMPs^13^. Thus, remission of acinar injury by Hspb1 could disturb the interplay between damaged acinar cells and inflammatory cells.

Apart from lytic cell death, programmed cell death, including apoptosis, autophagy, ferroptosis and necroptosis, is prevalent in SAP^47^. We did not detect any difference in autophagy or necroptosis affected by Hspb1 activity in acinar cells, but apoptosis and ferroptosis were significantly regulated by Hspb1. Ferroptosis, an iron-dependent type of cell death, is induced by trypsin and other pathological factors in acinar cells^48^. A previous report showed that ferroptosis increases the severity of AP and accelerates the development of chronic pancreatitis^26^. Cytologically, ferroptosis is characterized by the loss of mitochondrial cristae and the rupture and condensation of the mitochondrial membrane after lipid peroxidation^49^. A previous report showed that Hspb1 could inhibit erastin-induced ferroptosis by decreasing the iron-mediated production of lipid ROS^23^. These findings suggest the potential role of ferroptosis in AP and preliminarily show the relationship between Hspb1 and lipid ROS. Furthermore, our results demonstrated that Hspb1 reduced ferroptosis by increasing the expression of SLC3A2 and SLC7A11 and maintaining the antioxidative activity of Prdx1 in acinar cells.

We established a genetic CP model using mice with a p.D23A mutation in the trypsinogen 7 precursor. Previous studies suggested that the expression of R122H or p.D23A trypsinogen alone in genetic mouse models could directly cause AP^50^. A preclinical model of CP was established based on knock-in of the p.D23A mutation in the trypsinogen 7 precursor and crossing with a Cre strain that expresses Cre recombinase in the early mouse embryo (T7D23A strain)^29^. Similarly, in our CP model, the Tryp7^mut^ strain was bred with the E2a-Cre strain. T7D23A mice develop spontaneous AP at an early age, followed by progressive atrophic CP with acinar cell loss, fibrosis and adipose replacement. In addition to the histological evidence in the pancreas, many characteristics collectively indicate that our T7D23A mice are an ideal CP model, as indicated by an obvious loss of weight at 10 weeks of age, fatty diarrhea and hyperglycemia in the late stage accompanied by a marked reduction in amylase and lipase levels in plasma (data not shown). Moreover, SAP-associated diabetes is a complication of SAP characterized by persistent hyperglycemia and requires management through lifestyle changes, medication, and insulin therapy. At present, there is a lack of suitable mouse models that accurately replicate the natural progression of SAP-associated diabetes. The T7D23A strain provides a promising model for studying SAP-associated diabetes, exhibiting all stages of disease development and demonstrating hyperglycemia beginning at approximately week 10. Our report is the first to demonstrate that the T7D23A strain is a suitable model for exploring the molecular mechanism of CP.

In summary, our study revealed that Hspb1 is an essential suppressor of SAP in mice. Hspb1 inhibits acinar cell apoptosis and ferroptosis by eliminating ROS and lipid ROS. Mechanistically, Hspb1 interacts with Anxa2 to control its aggregation and phosphorylation and synergizes with Anxa2 to reduce the phosphorylation of Prdx1 and maintain its antioxidative activity. These findings provide new insights into the process of the progression of AP into SAP and suggest new therapeutic options for patients with this deadly disease.

## Supplementary Material

Supplementary figures.
