# Supplementary material for: Hspb1 protects against severe acute pancreatitis by attenuating apoptosis and ferroptosis via interacting with Anxa2 to restore the antioxidative activity of Prdx1
Source: Int J Biol Sci. 2024 Feb 25;20(5):1707–28. doi: 10.7150/ijbs.84494 (PMC10929186; doi:10.7150/ijbs.84494)
Supplement: Supplementary file 1 — Supplementary figures. [file ijbsv20p1707s1.pdf]

Supplementary Figure legends

Fig. S1 (A) PCA (Principal Component Analysis) of transcriptomic data from normal, AP and SAP C57BL/6 mouse samples. (B) Volcano plot showing the differentially expressed genes between AP model mice and normal controls and between SAP model mice and normal controls. (C) Hierarchical clustering analyses identified 4 major expression modules from the transcriptomic analysis. (D) KEGG analysis of the pathways enriched in SAP vs. AP using transcriptomic analysis. (E) PCA of proteomic data from normal, AP and SAP samples from C57BL/6 mice. (F) KEGG analysis of the enriched in the differentially expressed proteins between SAP and AP model mice using proteomic data. (G) Integrative analysis of transcriptomic and proteomic data and 1400 DEGs that coexisted between the AP vs. control group and 802 between the SAP vs. control group. (H) List of coexisting genes that were significantly upregulated in AP but downregulated in SAP. (I) List of coexisting genes that were significantly downregulated in AP but upregulated in SAP.

Fig. S2 (A) Representative images of Ly6G (green) in WT and Hspb1 KO mice generated by 8 hourly injections of caerulein. Scale bar, 50 μm. (B) Schematic of the process of establishing SAP models using mice via AAV8 administration. (C) Amylase and lipase activity in the plasma of the AAV8-con and AAV8-Hspb1 groups in the caerulein-induced SAP model. n=5. (D-E) Representative images and statistical analysis of Ly6G or CD68 (green) expression in the pancreas between the AAV8-ctrl and AAV8-Hspb1 groups in caerulein-induced SAP (n=5). Scale bar, 50 μm. (F) Flow cytometry analysis of blood parameters in the AAV8-ctrl and AAV8-Hspb1 groups in the duct ligation-induced SAP model. Ctrl, control. **p<0.01.

Fig. S3 (A) Representative images and statistical analysis of cleaved caspase 3 expression in the pancreas according to IHC staining between WT and Hspb1 KO mice with caerulein-induced AP (n=5). Scale bar, 100 μm. (B) Western blot analysis

and corresponding statistical analysis, comparing the expression of cleaved caspase 3 between WT and Hspb1 KO mice with caerulein-induced AP (n=5). (C) Representative images and statistical analysis of cleaved caspase 3 expression in the pancreas according to IHC staining in the AAV8-ctrl and AAV8-Hspb1 groups of patients with caerulein-induced SAP (n=5). Scale bar, 100 μm. (D) Western blot analysis and corresponding statistical analysis, comparing the expression of cleaved caspase 3 between the AAV8-ctrl and AAV8-Hspb1 mice with caerulein-induced AP (n=5). (E-G) Representative images of IHC staining, Western blot and statistical analysis of SLC3A2 and SLC7A11 expression in the pancreas between WT and Hspb1 KO mice with caerulein-induced AP (n=5). Scale bar, 100 μm. *$p<0.5$; ***$p<0.001$.

Fig. S4 (A-B) Representative images of IHC staining, Western blot and statistical analysis of Anxa2 expression in the pancreas between WT and Hspb1 KO mice with caerulein-induced AP (n=5). Scale bar, 100 μm. (C-D) Representative images of IHC staining, Western blot and statistical analysis of Anxa2 expression in the pancreas between the AAV8-ctrl and AAV8-Hspb1 groups in SAP patients (n=5). Scale bar, 100 μm. (E-F) Representative images of IHC staining, Western blot and statistical analysis of p-Anxa2 expression in the pancreas of normal, WT and Hspb1-KO mice with caerulein-induced AP (n=5). Scale bar, 100 μm. WT, wild type; KO, Anxa2 KO. *$p<0.5$, **$p<0.01$, ***$p<0.001$.

Fig. S5 (A) Schematic diagram describing the strategy used to establish Anxa2 KO mice. (B-C) H&E staining and statistical analysis were performed to evaluate the pancreatic tissue of WT and Anxa2 KO mice treated with or without AAV8-Hspb1 (n=5-6). Scale bar, 100 μm. (D) Amylase and lipase activity in the plasma of WT and Anxa2 KO mice treated with or without AAV8-Hspb1 (n=5-6). (E) 266-6 cells were pretreated as indicated. Representative images and statistical analysis of the fluorescence signal at a 594 nm wavelength after incubation with 5 μM DHE for 10

min. Scale bar, 50 μm. (F) Representative images and statistical analysis of the fluorescence signal at a 488 nm wavelength after incubation with 2.5 μM BODIPY 581/591 C11 for 30 min. Scale bar, 50 μm. NC, negative control; TAC, taurocholate acid; oe, overexpression; si, siRNA; WT, wild type; KO, Anxa2 KO. *p<0.5, N.S., not significant; ***p<0.001.

Fig. S6 (A-C) Representative images of IHC staining, Western blot and statistical analysis of pancreatic Prdx1 and p-Prdx1 expression in WT and Hspb1-KO mice with caerulein-induced AP (n=5). Scale bar, 100 μm. (D-F) Representative images of IHC staining, Western blot and statistical analysis of Prdx1 and p-Prdx1 expression in the pancreas in the AAV8-ctrl and AAV8-Hspb1 groups of SAP patients (n=5). Scale bar, 100 μm. *p<0.5, N.S., not significant; **p<0.01.

Fig. S1

A

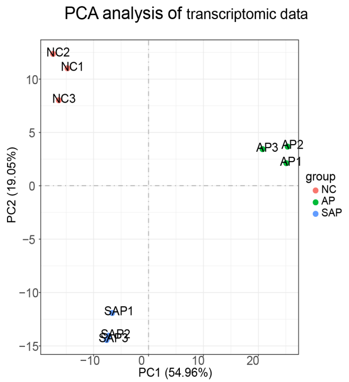

B

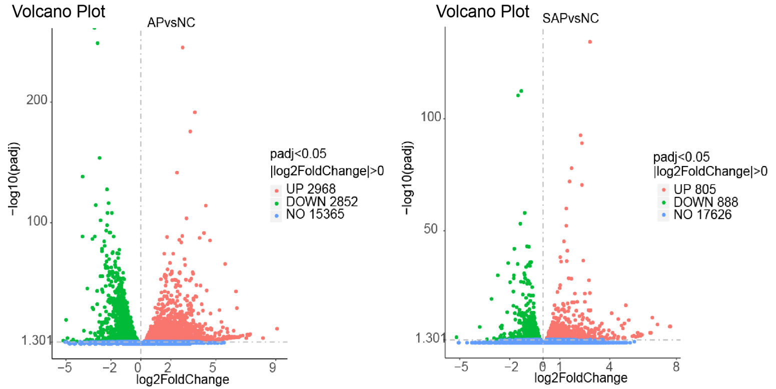

C

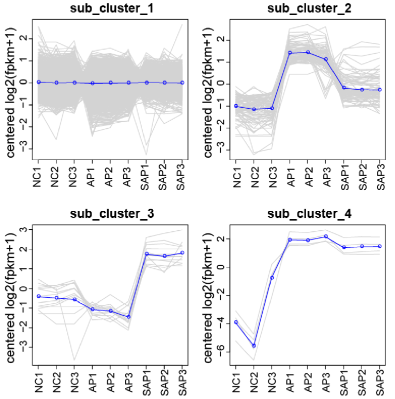

D

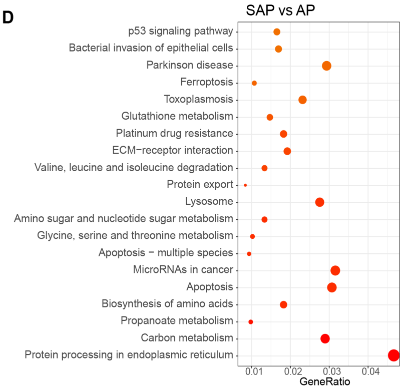

E

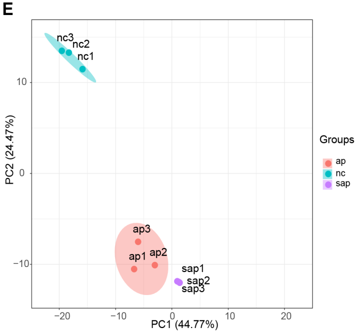

F

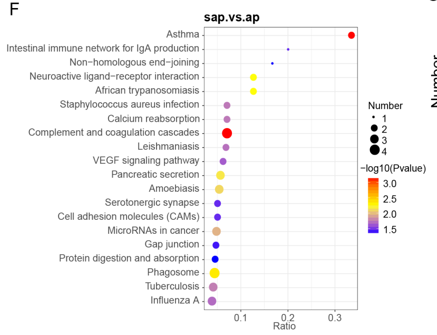

G

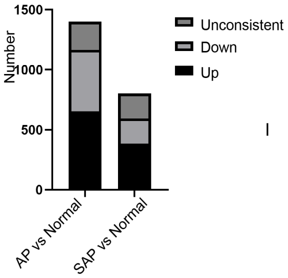

H

Gene name	Gene name	Gene name	Gene name
Pmm1	Ang	Sfn	Rbpms
Nrp1	Akr1b8	Phlda3	
Limd2	Rnase4	Map4	
Mettl27	Krt19	Cldn4	
Krt8	Fdft1	Clu	
Hmgcs1	Fads2	Hspb1	
Fhl3	Tax1bp3	Zyx	
Igfbp4	Stbd1	Krt18	

I

Gene name	Gene name	Gene name	Gene name	Gene name
Ppp4r4	Slc7a2	Agr2	Yipf6	Iigp1
Plekhf1	Rab3a	Hk2	Ighv7-1	6430548M08
Slc38a5	Susd2	Ifit1	Cox15	
Rab3d	Syvn1	Ggt1	Trabd	

Fig. S2

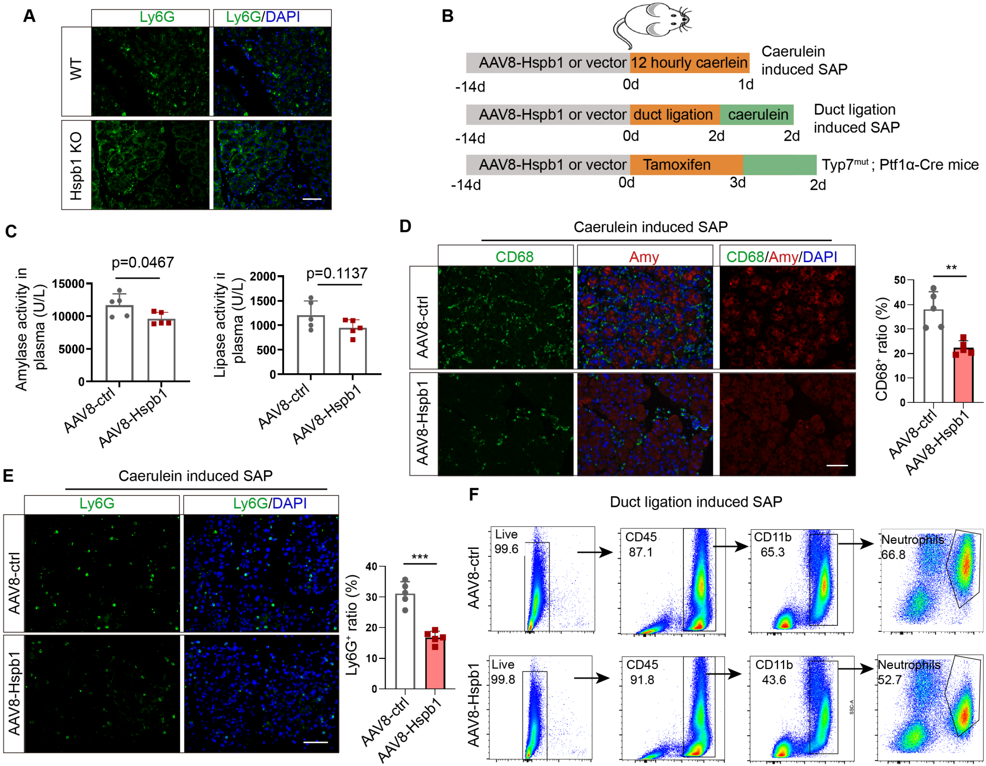

A
Ly6G
Ly6G/DAPI
WT
Hspb1 KO
B
AAV8-Hspb1 or vector
12 hourly caerlein
Caerulein induced SAP
-14d
0d
1d
AAV8-Hspb1 or vector
duct ligation
caerulein
Duct ligation induced SAP
-14d
0d
2d
2d
AAV8-Hspb1 or vector
Tamoxifen
Typ7mut ; Ptf1α-Cre mice
-14d
0d
3d
2d
C
p=0.0467
Amylase activity in plasma (U/L)
15000
10000
5000
0
AAV8-ctrl
AAV8-Hspb1
p=0.1137
Lipase activity in plasma (U/L)
2000
1500
1000
500
0
AAV8-ctrl
AAV8-Hspb1
D
Caerulein induced SAP
CD68
Amy
CD68/Amy/DAPI
AAV8-ctrl
AAV8-Hspb1
**
CD68+ ratio (%)
50
40
30
20
10
0
AAV8-ctrl
AAV8-Hspb1
E
Caerulein induced SAP
Ly6G
Ly6G/DAPI
AAV8-ctrl
AAV8-Hspb1
***
Ly6G+ ratio (%)
40
30
20
10
0
AAV8-ctrl
AAV8-Hspb1
F
Duct ligation induced SAP
AAV8-ctrl
Live 99.6
CD45 87.1
CD11b 65.3
Neutrophils 66.8
AAV8-Hspb1
Live 99.8
CD45 91.8
CD11b 43.6
Neutrophils 52.7

Fig. S3

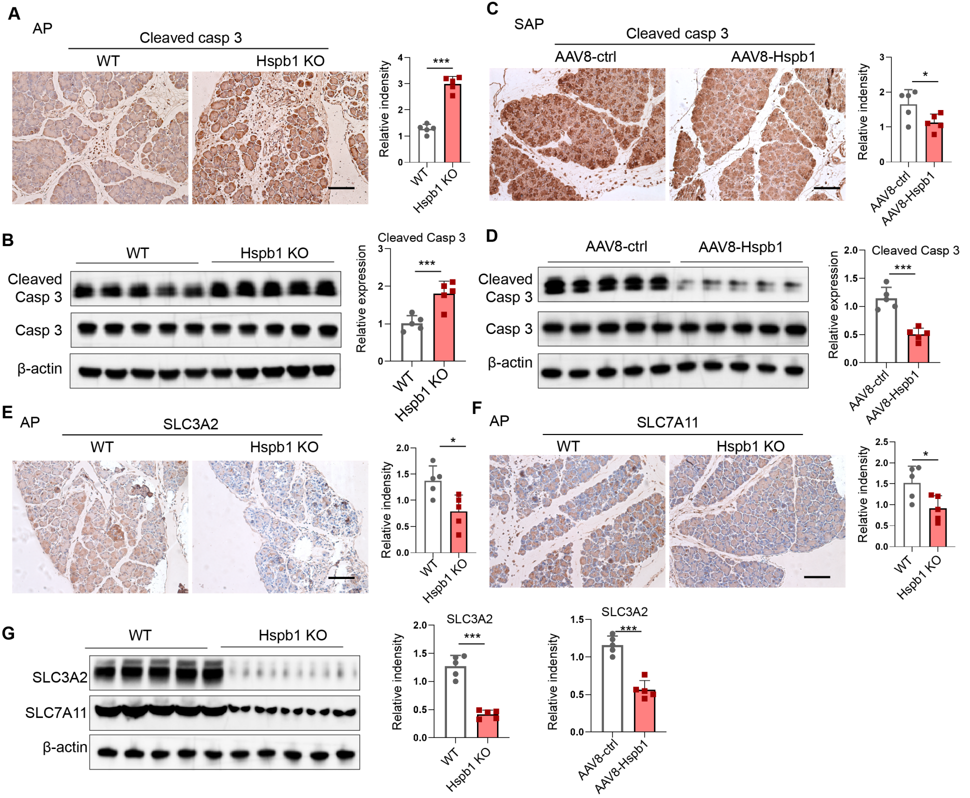

A
AP
Cleaved casp 3
WT
Hspb1 KO
Relative indensity
***
WT
Hspb1 KO
C
SAP
Cleaved casp 3
AAV8-ctrl
AAV8-Hspb1
Relative indensity
*
AAV8-ctrl
AAV8-Hspb1
B
WT
Hspb1 KO
Cleaved Casp 3
Casp 3
β-actin
Cleaved Casp 3
Relative expression
***
WT
Hspb1 KO
D
AAV8-ctrl
AAV8-Hspb1
Cleaved Casp 3
Casp 3
β-actin
Cleaved Casp 3
Relative expression
***
AAV8-ctrl
AAV8-Hspb1
E
AP
SLC3A2
WT
Hspb1 KO
Relative indensity
*
WT
Hspb1 KO
F
AP
SLC7A11
WT
Hspb1 KO
Relative indensity
*
WT
Hspb1 KO
G
WT
Hspb1 KO
SLC3A2
SLC7A11
β-actin
SLC3A2
Relative indensity
***
WT
Hspb1 KO
SLC3A2
Relative indensity
***
AAV8-ctrl
AAV8-Hspb1

Fig. S4

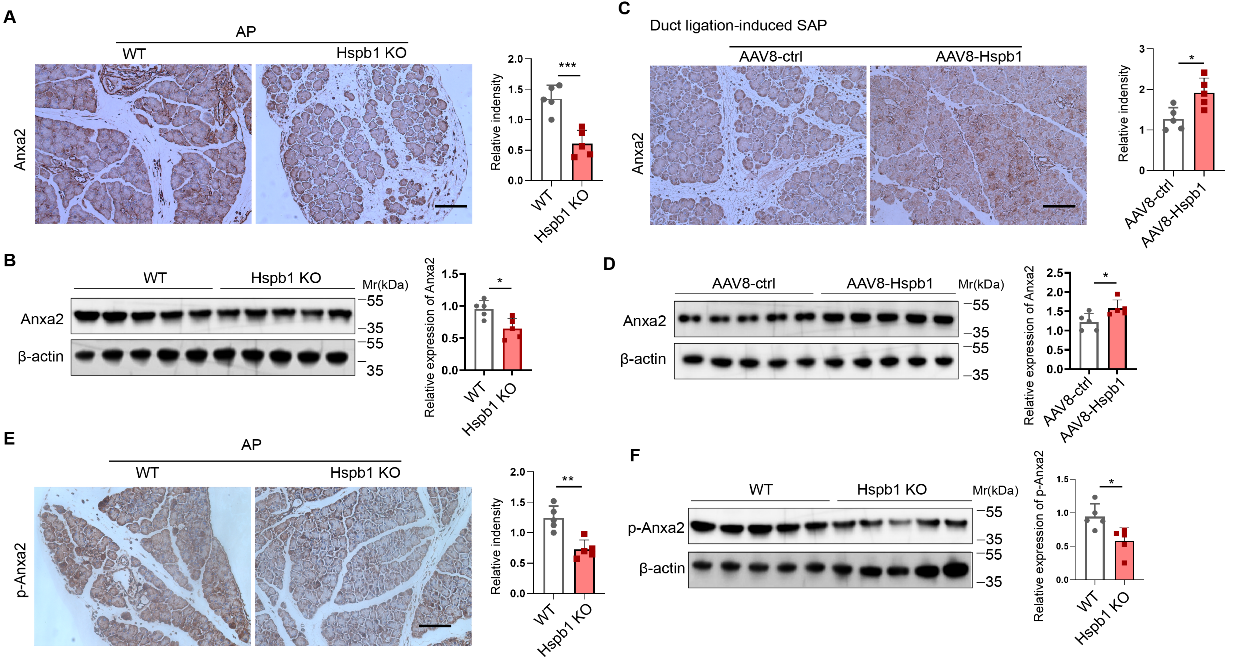
A
AP
WT
Hspb1 KO
Anxa2
Relative indensity
***
C
Duct ligation-induced SAP
AAV8-ctrl
AAV8-Hspb1
Anxa2
Relative indensity
*
B
WT
Hspb1 KO
Mr(kDa)
Anxa2
β-actin
55
35
Relative expression of Anxa2
*
D
AAV8-ctrl
AAV8-Hspb1
Mr(kDa)
Anxa2
β-actin
Relative expression of Anxa2
*
E
AP
WT
Hspb1 KO
p-Anxa2
Relative indensity
**
F
WT
Hspb1 KO
Mr(kDa)
p-Anxa2
β-actin
Relative expression of p-Anxa2
*

Fig. S5

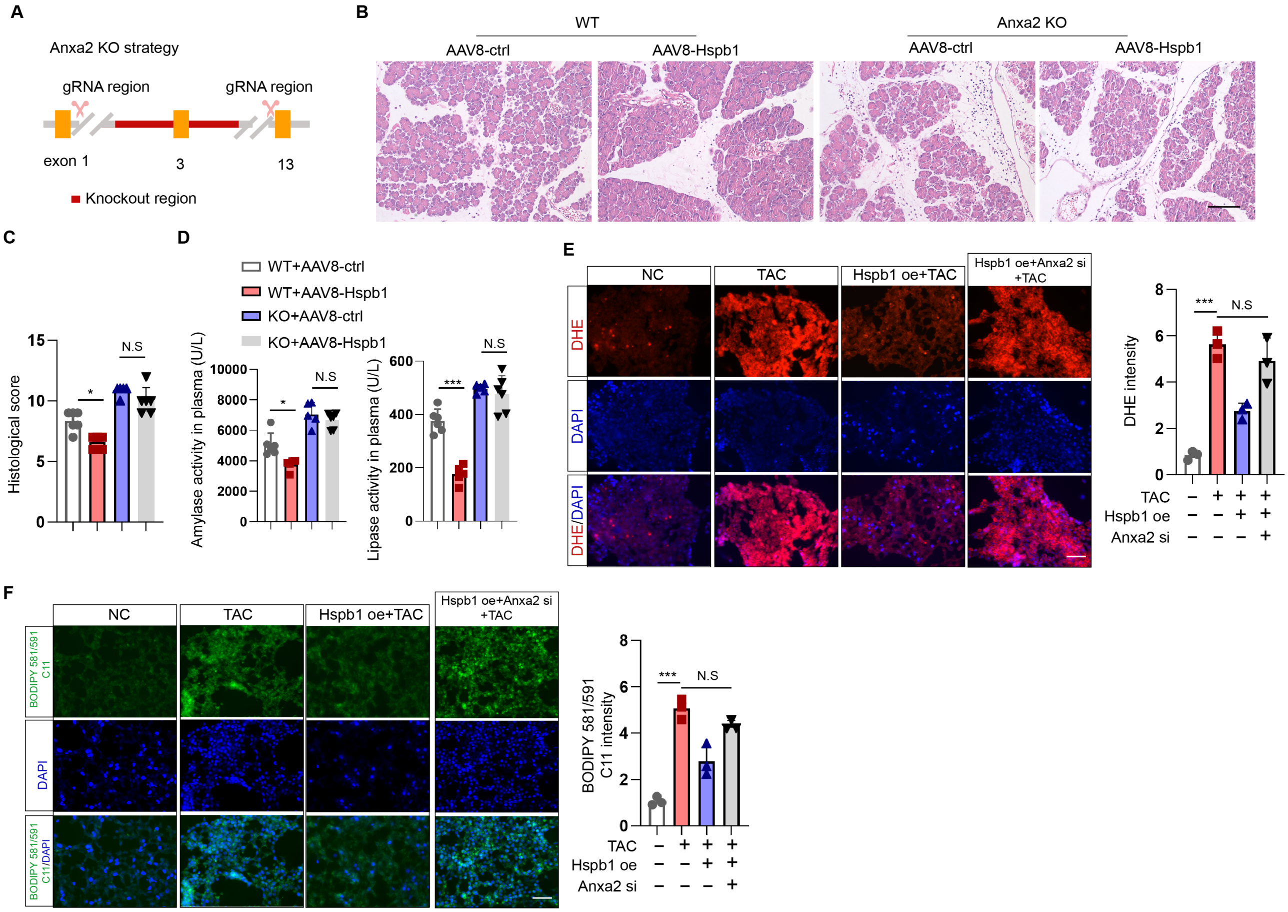
A
Anxa2 KO strategy
gRNA region
gRNA region
exon 1
3
13
Knockout region
B
WT
Anxa2 KO
AAV8-ctrl
AAV8-Hspb1
AAV8-ctrl
AAV8-Hspb1
C
Histological score
15
10
5
0
*
N.S
D
WT+AAV8-ctrl
WT+AAV8-Hspb1
KO+AAV8-ctrl
KO+AAV8-Hspb1
Amylase activity in plasma (U/L)
10000
8000
6000
4000
2000
0
*
N.S
Lipase activity in plasma (U/L)
600
400
200
0
***
N.S
E
NC
TAC
Hspb1 oe+TAC
Hspb1 oe+Anxa2 si +TAC
DHE
DAPI
DHE/DAPI
DHE intensity
8
6
4
2
0
***
N.S
TAC – + + +
Hspb1 oe – – + +
Anxa2 si – – – +
F
NC
TAC
Hspb1 oe+TAC
Hspb1 oe+Anxa2 si +TAC
BODIPY 581/591 C11
DAPI
BODIPY 581/591 C11/DAPI
BODIPY 581/591 C11 intensity
8
6
4
2
0
***
N.S
TAC – + + +
Hspb1 oe – – + +
Anxa2 si – – – +

Fig. S6

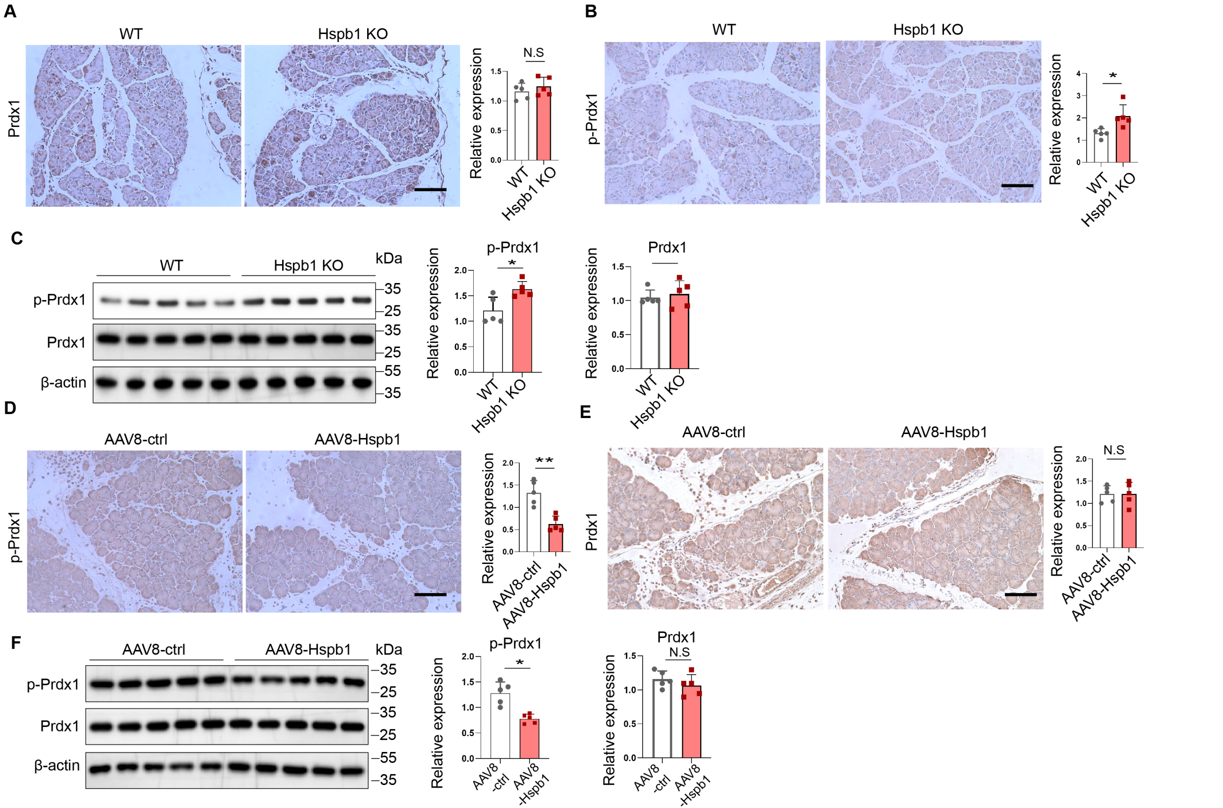
A
WT
Hspb1 KO
Prdx1
N.S
Relative expression
B
p-Prdx1
*
C
kDa
p-Prdx1
Prdx1
β-actin
35
25
55
D
AAV8-ctrl
AAV8-Hspb1
**
E
F
